# Co-expression of cancer-testis antigens of MAGE-A6 and MAGE-A11 is associated with tumor aggressiveness in patients with bladder cancer

**DOI:** 10.1038/s41598-021-04510-2

**Published:** 2022-01-12

**Authors:** Monireh Mohsenzadegan, Mahdieh Razmi, Somayeh Vafaei, Maryam Abolhasani, Zahra Madjd, Leili Saeednejad Zanjani, Laleh Sharifi

**Affiliations:** 1 Department of Medical Laboratory Science, Faculty of Allied Medical Sciences, Iran University of Medical Sciences (IUMS), Hemmat Highway, Tehran, Iran; 2 Oncopathology Research Center, Iran University of Medical Sciences (IUMS), Hemmat Street (Highway), Next To Milad Tower, 1449614535 Tehran, Iran; 3 Department of Molecular Medicine, Faculty of Advanced Technologies in Medicine, Iran University of Medical Sciences, Tehran, Iran; 4 Department of Pathology, School of Medicine, Iran University of Medical Sciences, Tehran, Iran; 5 Hasheminejad Kidney Center, Iran University of Medical Sciences, (IUMS), Tehran, Iran; 6 Uro‑Oncology Research Center, Tehran University of Medical Sciences (TUMS), Tehran, Iran

**Keywords:** Cancer, Cell biology

## Abstract

Melanoma antigen gene (MAGE)-A6 and MAGE-A11 are two of the most cancer-testis antigens overexpressed in various types of cancers. However, the clinical and prognosis value of MAGE-A6 and MAGE-A11 co-expression in the pathophysiology of the bladder is unknown. Three studies were selected from GEO databases in order to introduce the common genes that are involved in bladder cancer. Then immunohistochemical analysis for staining pattern and clinicopathological significance of suggested markers, MAGE-A6 and MAGE-A11, were performed in 199 and 213 paraffin-embedded bladder cancer with long adjacent normal tissues, respectively. A significant and positive correlation was found between both nuclear and cytoplasmic expressions of MAGE-A6 as well as expression of cytoplasmic MAGE-A11 with histological grade, PT stage, lamina propria invasion, and LP/ muscularis (L/M) involvement (all of the p-values in terms of H-score were < 0.0001). Additionally, significant differences were found between both nuclear and cytoplasmic MAGE-A6/MAGE-A11 phenotypes with tumor size (P = 0.007, P = 0.043, respectively), different histological grades, PT stage, LP involvement, and L/M involvement (all of the p-values for both phenotypes were < 0.0001). The current study added the value of these novel markers to the bladder cancer clinical settlement that might be considered as an admirable target for immunotherapy.

## Introduction

Bladder cancer (BC) is considered the most frequent malignancy of the genitourinary tract worldwide, with an estimated 549,000 new cases and 200,000 deaths annually^1,2^. Urothelial cell carcinoma is the principal histological type of BC, which is also called transitional cell carcinoma (TCC), categorized into non-muscle-invasive BC (NMIBC) and muscle-invasive BC (MIBC) based on the presence of tumor invasion into the muscularis propria^3^. According to the American Cancer Society, an estimated 83,730 new cases (64,280 in men and 19,450 infemales) and 17,200 deaths (12,260 in men and 4,940 in females) from bladder cancer occur in 2021^4^. Despite diagnosing only approximately 10–30% of new cases with the muscle-invasive disease, MIBC has accounted for the main reason for reduced long-term survival^5^. In spite of important advances in the surgical techniques and therapeutic approaches, BC continues to pose a profound challenge to clinicians owing to a high rate of recurrence within 5 years after therapy and a great probability of progression to aggressive, muscle-invasive, and metastatic forms^6,7^. Of note, biomarkers have become valuable promising tools for improving and optimizing early-stage diagnosis, high-risk patient stratification, clinical management, and prognosis of BC. Nevertheless, the disease burden still remains high with a remarkable unsatisfactory prognosis^8,9^. Therefore, the identification of novel robust biomarkers for precise diagnosis and prognosis as well as specific targeted therapy is urgently warranted to improve BC surveillance in clinical settings^10^.

Based on three studies reporting on BC (gene expression profile of GSE6161615, GSE27448, and GSE100926 from GEO database), we became particularly interested in melanoma antigens genes-A6 (MAGE-A6) and melanoma antigens genes-A11 (MAGE-A11). Bioinformatics analysis evaluation was introduced as an innovative novel approach in the field of biomarker discovery with relatively limited resources^11^. In fact, this progress relies on an interplay between high throughput experimentation and analysis technologies that can be applied in molecular pathology^12^. In the current study, protein–protein interaction (PPI) network analysis was performed for output data of available BC tissue samples in comparison to control tissues. We tried to apply some online analysis for a better understanding of the genes in the related network. It was found that MAGE-A11 and MAGE-A6 could be practical markers in BC.

Nowadays, a growing number of studies reported by our and other groups illustrate the MAGE-A antigens as promising prognostic markers and appropriate targets for cancer immunotherapy, owing to their involvement in a wide range of oncogenic procedures^13–16^. The MAGE-A family proteins belong to the cancer-testis antigens (CTA) group, whose expression is typically limited to male germ cells but is de-repressed in a broad spectrum of human tumors^17^. The tumor-specific expression of MAGE-A proteins resulted in various clinical immunotherapy trials targeting MAGE-A antigens^18,19^. The immunogenicity of MAGE-A antigens in patients with cancer has made them an attractive candidate for cancer immunotherapy or vaccination in solid tumors^19^. In addition to their importance in cancer immunotherapy, MAGE-A proteins have been identified to participate in tumor progression as oncoproteins^20^. Particularly, MAGE-A proteins bind directly to the RING family of ubiquitin E3 ligase, regulate the activity of E3 ubiquitin ligase, and promote the ubiquitin-dependent degradation of various tumor suppressors, such as p53 and AMPKα1, thus aiding in the tumorigenesis and aggressively growing cancer cells. Additionally, MAGE-A proteins act as transcriptional co-regulators in the progression of tumors through interaction with transcription factors^21^.

Recent reports have cleared that MAGE-A11, as one of the MAGE family members, is a proto-oncogene whose elevated expression affects various signaling pathways involved in tumor growth and progression^22^. MAGE-A11 has been found to form a complex with androgen receptor (AR), resulting in enhanced transcriptional activity of human AR. The MAGE-A11 overexpression promotes the development of prostate cancer through increasing AR signaling^23^. In addition, MAGE-A11 is involved in transcriptional activation of progesterone receptor (PR)^17^. Consistent with a function in cancer progression, high expression of MAGE-A11protein has been found to be associated with higher stages and worse prognosis in multiple tumor lineages, such as head and neck squamous cell carcinoma^24^, breast cancer^25^, and esophageal carcinoma^26^. Additionally, it has been proposed that specific subgroups of MAGE-A members have the functional collaboration to potentiate specific oncogenic functions. Significant to this issue, Julieta E. Laiseca’s group has reported that MAGE-A11 and MAGE-A6 form a protein complex leading to the MAGE-A11 stabilization and consequently the AR activity augmentation and promote tumor progression in prostate cancer^27^. Moreover, MAGE-A6 expression could also serve as a cancer prognostic marker, based on previous data showing that MAGE-A6 was correlated with tumor progression and reduced survival^28,29^.

While the aforementioned evidence illustrated that MAGE-A11 may potentiate cancer development at least in part through the functional collaboration with MAGE-A6, the clinical value of the MAGE-A11 expression in association with MAGE-A6 expression has not been fully elucidated. Therefore, the current study was designed, for the first time, to explore the expression pattern, potential clinical significance, and the relationship between MAGE-A11 and MAGE-A6 in a series of BC tissues through immunohistochemistry (IHC) technique on tissue microarray (TMA) slides.

## Results

### Bioinformatics approach

**Figure 1:**
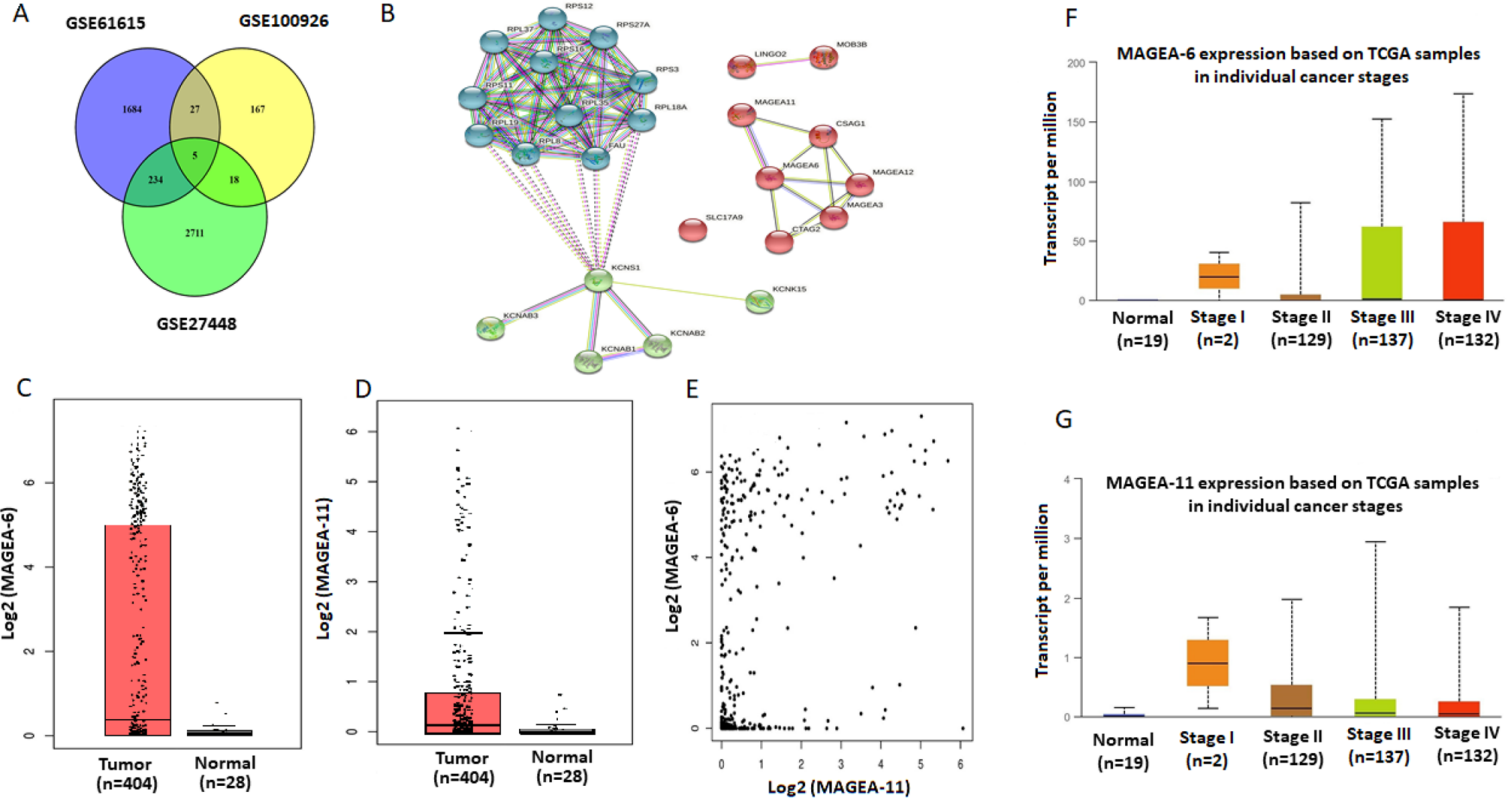
Bioinformatic analysis of bladder cancer studies in online different databases. (**A**) The Venn diagram by Venny (https://bioinfogp.cnb.csic.es/tools/venny/index2.0.2.html)^66^ represents the overlaps of differential protein expressions between three subtypes of BC. Five common differential expressions genes (LINGO2, SLC17A9, KCNS1, MAGEA6, and MAGEA11) were obtained from the GEO database, including GSE61615, GSE27448, and GSE100926. **(B)** PPI via STRING (https://string-db.org/)^69^ of common differential protein expressions was obtained by the string with the highest degree of connectivity (highest confidence > 0.9) in three GEO databases of BC. **(C)** The Box plot expression results of the BC Match TCGA normal and GTEx data showed that increased expression of MAGE-A6 protein on GEPIA database (p < 0.05, |Log2FC| Cutoff: 2) (http://gepia.cancerpku.cn/index.html)^73^. **(D)** The Box plot expression results of the BC Match TCGA normal and GTEx data showed that increased expression of MAGE-A11 protein on GEPIA database (p < 0.05, |Log2FC| Cutoff: 2) . (E) The Co-expression results of genes predicted by the GEPIA database online analysis showed that there is a statistically significant spearman correlation between MAGE-A6 and MAGE-A11 protein in BC and normal based on TCGA samples (Log-rank test; P = 4e-23). **(F)** Box plot analysis of the relative expression levels of MAGE-A6 in normal tissues and BC patient’s individual cancer stage (I-IV) tissues by UALCAN database (http://ualcan.path.uab.edu/)^72^. Based on the standard definitions, each box-plot shows the median (bold line) and interquartile lines (box). The result of Mann–Whitney U test showed that there is an association for the median of expression between Normal-vs-Stage2 (p = 2.541000E-03), Normal-vs-Stage3 (p = 4.42790000000359E-05), Normal-vs-Stage4 (p = 1.189250E-04), Stage2-vs-Stage3 (p = 1.790020E-01), Stage2-vs-Stage4 (p = 2.900000E-01), Stage3-vs-Stage4 (p = 7.813600E-01), and there were no statistically significant differences in the median level of MAGE-A6 mRNA expression between Stage1-vs- Normal and the other stages. **(G)** Box plot analysis of the relative expression levels of MAGE-A11 in normal tissues and BC patient’s individual cancer stage (I-IV) tissues by UALCAN database. Based on the standard definitions, each box-plot shows the median (bold line) and interquartile lines (box). The result of Mann–Whitney U test showed that there is an association for the median of expression between Normal-vs-Stage1 (p = 4.896000E-01), Normal-vs-Stage2 (p = 2.821200E-03), Normal-vs-Stage3 (p = 1.76258999999845E-05), Normal-vs-Stage4 (p = 4.155100E-04), Stage1-vs-Stage2 (p = 7.867800E-01), Stage1-vs-Stage3 (p = 6.622000E-01), Stage1-vs-Stage4 (p = 7.758200E-01), Stage2-vs-Stage3 (p = 2.979400E-01), Stage2-vs-Stage4 (p = 5.875000E-01), Stage3-vs-Stage4 (p = 7.793800E-02). BC: bladder cancer, GEPIA: gene expression profiling interactive analysis web server, GTEx: genotype-tissue expression project PPI: protein protein interaction, TCGA: the cancer genome atlas.

Three studies GSE27448^30–32^, GSE100926^33^, and GSE61615^34^ that each included the information of BC tissue and control tissues were explored. GSE27448 included: 1) GSE89figure dataset (GDS183), comprised of 40 BC samples; 2) GSE3167 dataset (GDS1479), comprised of 60 samples (9 controls and 51 BC samples); 3) GSE7476 dataset, composed of 12 samples (3 controls and 9 bladder cancer samples) and 4) GSE12630 dataset, comprised of 19 BC samples. In total, their pooled microarray analysis was composed of 17 control samples and 129 BC samples. GSE100926 consisted of three controls and three BC samples and GSE61615 consisted of two controls and two BC samples. The results were analyzed and the statistically significant differential expression of genes in tumor tissues in comparison to control tissues was obtained from selected previous studies (P < 0.05*,* supplementary Table 1). Venn diagram analysis was performed to find common significant differential in this GSE BC mentioned (Fig. 1A).

Our list was the LINGO2 (Leucine-Rich Repeat and Ig Domain Containing 2), SLC17A9 (Solute Carrier Family 17 Member 9), KCNS1 (Potassium Voltage-Gated Channel Modifier Subfamily S Member 1), MAGEA6, and MAGEA11. LINGO2 encodes a transmembrane protein in addition to its role in Parkinson 's disease^35^, identified as a cancer stem cell-associated protein in gastric cancer initiation and progression by altering cell motility, stamens, and tumorigenicity both in vitro and in patient-derived tissues^36^. SLC17A9 can be used as a new molecular marker to predict the poor prognosis of patients with hepatocellular carcinoma^37^. It is also may play a role in the progression of colorectal cancer^38^ and may potentially be used as an independent biomarker for gastric carcinoma prognostic evaluation as well^39,40^. KCNS1 was reported as a bone metastasis signature using a supervised classification approach in a large series of breast cancer patients^41^ and variations in this potassium channel genes were associated with the occurrence of preoperative breast pain^42^. Accumulating data proved that MAGE-A11 contributes to the genetic susceptibility and prognosis for renal cell carcinoma as a biomarker for occurrence and prognosis^43^. Besides, studies underscore that MAGE-A11 expression has a negative predictive role in head and neck cancer^44,45^ and valuable diagnostic or prognostic marker as well as a potential molecular therapeutic target^46^. This gene not only portrays DNA hypermethylation but also is important in histone deacetylation for the mechanism underlying gene silencing in breast cancer patients^25,47,48^. Additionally, more research was conducted in identifying new approaches for developing related to the clustered MAGE-A expression analysis cancer-specific therapeutics in esophageal squamous cell carcinoma (ESCC)^49,50^ and prostate cancer as well^22,51^. Overall we intend to focus on MAGE family members in BC for the first time and provide their involvement in the development of new cancer treatment strategies.

Common genes with the highest degree of connectivity of PPI network analysis (highest confidence > 0.9) were identified (Fig. 1B). Gene ontology (GO) analysis of these common genes were included on Enrichr and their related GO and Reactome pathways were shown in Supplementary Table 1. In our evaluation, potassium channel complex among cellular components, purine/adenosin nucleotide transmembrane transporter activity, ATP/ADP transmembrane transporter activity, potassium channel regulator activity between molecular function were spotlighted. In addition to the above-mentioned activities, regulation of autophagy and cellular catabolic process throughout the biological process were considered significantly highlighted as well. It was shown that potassium channels regulate membrane potential, ion homeostasis, and electric signaling^52,53^. Furthermore, the presence and also activity of ion pumps/ channels has correlation with the cancer development via its proliferation, differentiation, apoptosis, and migration^54^.

In cBioportal, our genes among copy number alterations (CNA) and mutations of BC (12 studies, 2410 samples) were checked. In addition, a confirmatory analysis was conducted using the gene expression profiling interactive analysis (GEPIA) database to acquire more reliable analytic results related to tumor/control differential expression (Fig. 1C,D), and correlation analysis (Fig. 1E). MAGEA6 and MAGEA11 through UALCAN database based on cancer genome atlas (TCGA) were reported (Fig. 1F,G). Next, our genes were shown in BC (Supplementary Fig. 1). Finally, these two genes were selected for evaluation of expression using the IHC method in bladder tissues. Additionally, it was cleared that these two genes are expressed high, medium and low in the BC/ bladder tumor lines (Supplementary Fig. 2).

### Characteristics of study population

To evaluate the MAGE-A6 and MAGE-A11 expressions and their clinical relevance, the expression of these markers was determined in 199 and 213 BC tissues, respectively. Overall, the median age of the study population both MAGE-A6 and MAGE-A11 expression was 67 years (range 20–95). The study population consisted of 156 (78.4%) male and 43 (21.6%), female patients, with a male/female ratio of 3.6 for MAGE-A6 expression. For MAGE-A11 expression was 170 (80%) male and 43 (20%) female patients, with a male/female ratio of 3.9. This ratio is consistent with the prevalence of BC in men to women, estimated at 2: 1 to 4: 1^55^. Tumor size (at the largest diameter) ranged from 1 to 13 cm. Based on mean tumor size (2.5 cm), tumors were categorized into two groups both for MAGE-A6 and MAGE-A11 expression. Pathological and clinical data of patients and tumor characteristics are shown in Tables 1 and 2.

**Table 1:** Association between MAGE-A6 expressions (staining intensity and H-score) and clinic-pathological parameters of BC cases (P-value, Pearson’s chi-square test).

Patients and tumor characteristics	Total samples N (%)	Nuclear expression of MAGE-A6		Cytoplasmic expression of MAGE-A6	
		Staining Intensity	H-score	Staining Intensity	H-score
**Median age**					
**Years**					
≤ 67	95 (48)	**0.038**	**0.016**	0.563	0.932
> 67	104 (52)				
**Gender**					
Male	156 (78.4)	0.45	0.694	0.357	0.372
Female	43 (21.6)				
**Mean tumor size (cm)**					
≤ 2.5	127 (64)	0.112	**0.044**	0.185	0.125
> 2.5	72 (36)				
**Histological grade**					
Low	88 (44)	** < 0.0001**	** < 0.0001**	** < 0.0001**	** < 0.0001**
High	111 (56)				
**pT stage**					
pTa	86 (43.2)	** < 0.0001**	** < 0.0001**	**0.001**	** < 0.0001**
pT1	82 (41.2)				
pT2	31 (15.6)				
pT3	0 (0)				
pT4	0 (0)				
**Lamina propria involvement**					
Involved	113 (57)	** < 0.0001**	** < 0.0001**	** < 0.0001**	** < 0.0001**
None	86 (43)				
**Muscularis invasion**					
Involved	32 (16)	0.376	0.155	0.477	0.684
None	167 (84)				
**lamina propria/muscularis involvement (L/M)**					
L − /M-	86 (43.2)	** < 0.0001**	** < 0.0001**	**0.0001**	** < 0.0001**
L + /M-	82 (41.2)				
L + /M +	31 (15.6)				
**Recurrence**					
Present	52 (26)	0.517	0.661	0.545	0.695
Absent	147 (74)				
**Distant metastasis**					
Present	29 (14.6)	0.061	0.564	0.185	0.932
Absent	170 (85.4)				

Bold numbers represent significant p-values.

**Table 2:** Association between MAGE-A11 expressions (staining intensity and H-score) and clinic-pathological parameters of BC cases (P-value, Pearson’s chi-square test).

Patients and tumor characteristics	Total samples N (%)	Nuclear expression of MAGE-A11		Cytoplasmic expression of MAGE-A11	
		Staining Intensity	H-score	Staining Intensity	H-score
**Median age**					
**Years**					
≤ 67	108 (51)	0.079	0.075	0.638	0.932
> 67	105 (49)				
**Gender**					
Male	170 (80)	0.708	0.839	0.603	0.372
Female	43 (20)				
**Mean tumor size (cm)**					
≤ 2.5	134 (63)	0.741	0.789	0.139	0.125
> 2.5	79 (37)				
**Histological grade**					
Low	94 (44)	0.349	0.554	** < 0.0001**	** < 0.0001**
High	119 (56)				
**pT stage**					
pTa	87 (40.8)	0.219	0.579	** < 0.0001**	** < 0.0001**
pT1	95 (44.6)				
pT2	31 (14.6)				
pT3	0 (0)				
pT4	0 (0)				
**Lamina propria involvement**					
Involved	126 (59)	0.292	0.954	** < 0.0001**	** < 0.0001**
None	87 (41)				
**Muscularis invasion**					
Involved	31 (14.6)	0.14	0.339	0.334	0.684
None	182 (85.4)				
**Lamina propria/muscularis involvement (L/M)**					
L − /M-	87 (40.8)	0.219	0.579	**0.0001**	** < 0.0001**
L + /M-	95 (44.6)				
L + /M +	31 (41.6)				
**Recurrence**					
Present	57 (27)	0.577	0.692	0.203	0.695
Absent	156 (73)				
**Distant metastasis**					
Present	33 (15.5)	0.262	0.49	0.097	0.932
Absent	180 (84.5)				

Bold numbers represent significant p-values.

### MAGE-A6 and MAGE-A11 expressions in the BC and their association with clinicopathological parameters

**Figure 2:**
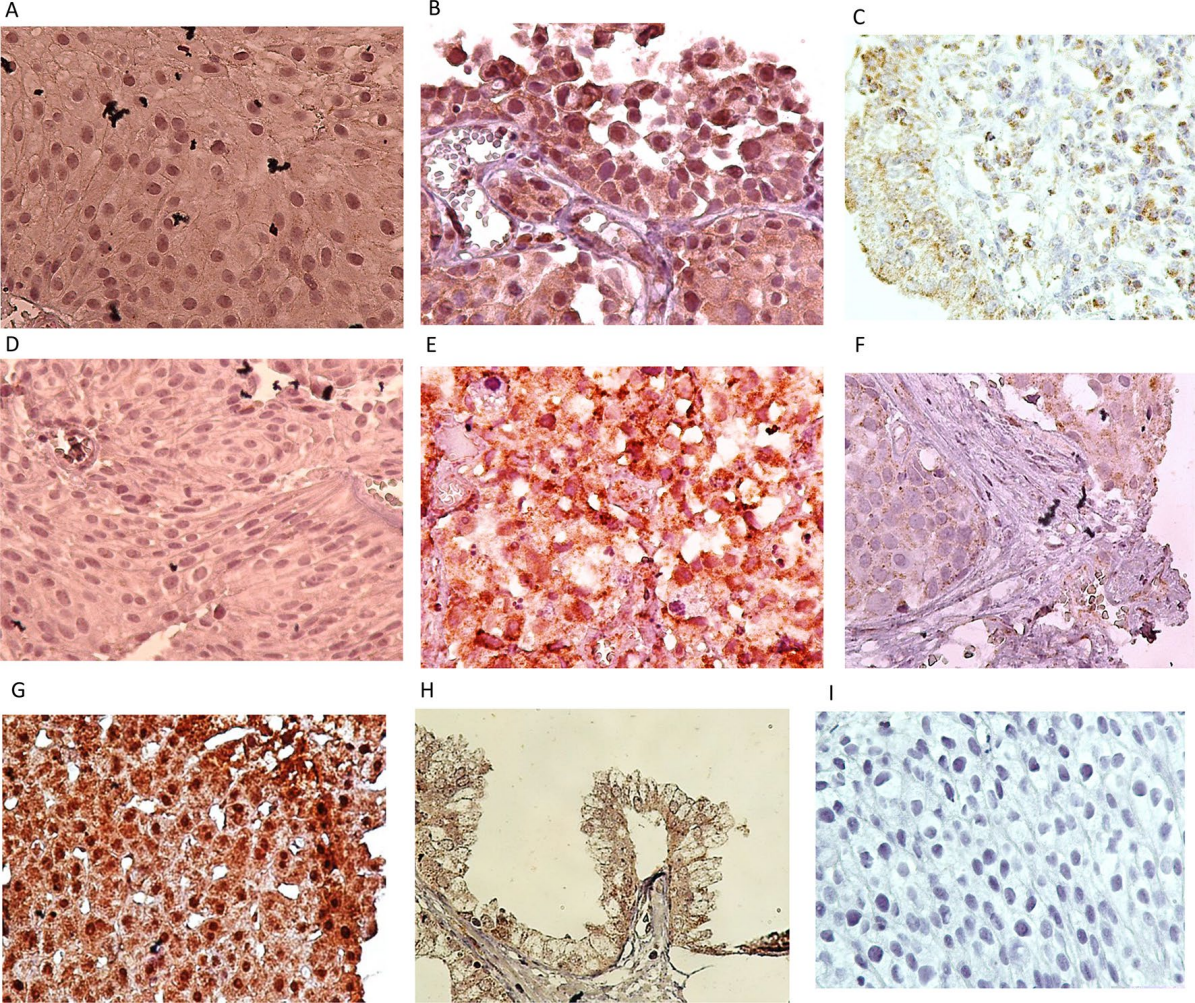
Staining pattern of MAGE-A6 expression **(A-C)** and MAGE-A11 expression **(D-F)** in bladder tissues. **(A).** Intermediate staining for both nuclear and cytoplasmic expressions in low-grade BC (pTa stage), **(B).** Strong staining for nuclear expression and intermediate staining for cytoplasmic expression in high-grade BC (pT1 stage), **(C).** MAGE-A6 expression in adjacent normal tissue, **(D).** Weak staining for both nuclear and cytoplasmic expressions in low-grade BC (pTa stage), **(E).** Strong staining for both nuclear and cytoplasmic expressions in high-grade BC (pT2 stage), **(F).** MAGE-A11 expression in adjacent non-tumoral tissue, **(G).** MAGE-A6 expression in liver tissue as a positive control, **(H).** MAGE-A11 expression in prostate tissue as a positive control, and **(I).** Staining of bladder tissue with a nonreactive antibody (anti-CD11b antibody, negative control). All images were taken at 400 × magnification.

The immunohistochemical analysis was performed to evaluate the expression of MAGE-A6 and MAGE-A11 in BC. Both MAGE-A6 (Fig. 2A–C) and MAGE-A11 (Fig. 2D–F) proteins were predominantly expressed in both the cytoplasm and the nucleus of bladder tumor cells. However, a considerable portion of the cancer tissues was negative for expression of nuclear MAGE-A11, while the majority of tissues were positive for expression of cytoplasmic MAGE-A11. No significant staining of MAGE-A6 and MAGE-A11 expressions were observed in the stroma (Fig. 2). The nuclear and cytoplasmic patterns had a variety of staining intensities in bladder tumor cells.

Of 199 bladder cases stained for nuclear MAGE-A6 expression; negative, weak, intermediate, and strong intensities were observed in 58(29.1%), 32 (16.1%), 21 (10.6%), and 88 (44.2%) of cases, respectively. For cytoplasmic expression, negative, weak, intermediate, and strong intensities were observed in 1(0.5%), 37 (18.6%), 107 (53.8%), and 54 (27.1) of cases, respectively (Table 3). The mean of nuclear MAGE-A6 H-score was 157 for cancerous tissue vs 11 for normal tissues and a strong significant was found between normal and cancerous tissues (P < 0.0001). Of 213 bladder cases stained for nuclear MAGE-A11, negative, weak, intermediate, and strong intensities were observed in 141(66.2%), 30 (14.1%), 24 (11.3%), and 18 (5.8%) of cases, respectively. For cytoplasmic expression, negative, weak, intermediate, and strong intensities were observed in 1(0.5%), 52(24.4%), 68 (31.9%), and 92 (43.2%) of cases, respectively (Table 3).

**Table 3:** MAGE-A6 and MAGE-A11 expression (Intensity of staining and H-score) in BC.

Scoring system	Bladder carcinoma	
	MAGE-A6	MAGE-A11
Intensity of staining of nuclear expression;	Samples N (%);	Samples N (%);
Negative (0)	58 (29.1)	141 (66.2)
Weak (+ 1)	32 (16.1)	30 (14.1)
Moderate (+ 2)	21 (10.6)	24 (11.3)
Strong (+ 3)	88 (44.2)	18 (8.5)
H-score;		
Low (1–100)	93 (46.7)	172 (80.8)
Moderate (101–200)	21 (10.6)	25 (11.7)
High (201–300)	85 (42.7)	16 (7.5)
Total	199	213
Intensity of staining of cytoplasmic expression;		
Negative (0)	1 (0.5)	1 (0.5)
Weak (+ 1)	37 (18.6)	52 (24.4)
Moderate (+ 2)	107 (53.8)	68 (31.9)
Strong (+ 3)	54 (27.1)	92 (43.2)
H-score;		
Low (1–100)	38 (19.1)	57 (26.8)
Moderate (101–200)	107 (53.8)	67 (31.5)
High (201–300)	54 (27.1)	89 (41.8)
Total	199	213

Significant differences were found between nuclear MAGE-A6 expression with age (P = 0.038), different histological grades (P < 0.0001), PT stage (P < 0.0001), LP involvement (P < 0.0001), and lamina propria / muscularis (L/M) involvement (P < 0.0001) in terms of intensity of staining. In this regard, significant differences were observed between nuclear MAGE-A6 expression with age (P = 0.016), tumor size (P = 0.044), different histological grade (P < 0.0001), PT stage (P < 0.0001), LP involvement (P < 0.0001), and L/M involvement (P < 0.0001) in terms of H-score (Table 1). Pearson’s χ2 analysis showed that there was a direct and positive relationship between the mentioned parameters with nuclear MAGE-A6 expression. As age, tumor size, histological grade, tumor invasion to LP, and L/M increased, nuclear MAGE-A6 expression increased. Significant differences were also found between cytoplasmic MAGE-A6 expression with different histological grades (P < 0.0001), PT stage (P < 0.0001), LP involvement (P < 0.0001) both in terms of intensity of staining and H-score. P values of intensity of staining and H-score for L/M involvement were 0.0001 and < 0.0001, respectively (Table 1). Pearson’s χ^2^ analysis showed that there was a direct and positive relationship between the mentioned parameters with cytoplasmic MAGE-A6 expression. As a histological grade, PT stage, tumor invasion to LP, and L/M increased, cytoplasmic MAGE-A6 expression increased. These results indicate an increase in both nuclear and cytoplasmic MAGE-A6 expression in advanced stages of BC.

No significant differences were found between nuclear MAGE-A11 expressions with clinicopathological parameters (Table 2). Significant differences were found between cytoplasmic MAGE-A11 expression with different histological grades (P < 0.0001), PT stage (P < 0.0001), LP involvement (P < 0.0001) both in terms of intensity of staining and H-score. P values of intensity of staining and H-score for L/M involvement were 0.0001 and < 0.0001, respectively (Table 2). Pearson’s χ^2^ analysis showed that there was a direct and positive relationship between the mentioned parameters with cytoplasmic MAGE-A11 expression. As a histological grade, PT stage, tumor invasion to LP and L/M increased, MAGE-A11 expression increased. These results indicate increased expression of cytoplasmic MAGE-A11 in advanced stages of BC.

**Figure 3:**
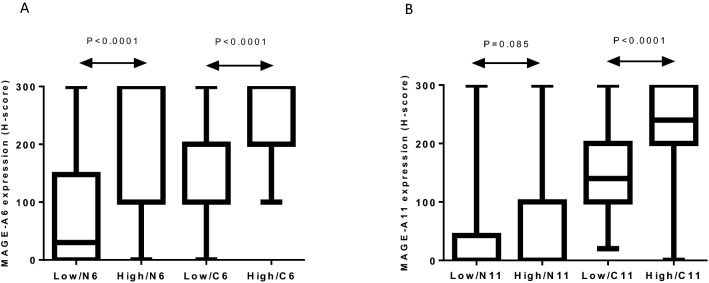
Differences of expression of MAGE-A6 **(A)** and MAGE-A11 **(B)** in low grades vs high grades of BC samples with immunohistochemical analysis (Mann–Whitney *U* test). C6: cytoplasmic expression of MAGE-A6, C11: cytoplasmic expression of MAGE-A11, high: high grade, low: low grade, N6: nuclear expression of MAGE-A6, N11: nuclear expression of MAGE-A11, P: p-value. Charts were drawn by Prism version 8.3.0 software (Graph Pad Inc., San Diego, CA, USA). https://www.graphpad.com/support/faq/prism-830-release-notes/.

Further analysis based on the Mann–Whitney U test showed a significant difference between both nuclear and cytoplasmic MAGE-A6 expressions with histological grade (P < 0.0001), such that in high grades, an increased expression was observed compared to low grades (Fig. 3A). In addition, there was a significant difference between the cytoplasmic MAGE-A11 expression and histological grade (P < 0.0001, Fig. 3B).

### Prognostic significance of MAGE‑A6 and MAGE-A11 expressions in terms of H‑score in BC

Of the 199 bladder tissues stained for MAGE-A6 and 213 tissues stained for MAGE-A11, metastasis and recurrence for tissues stained with MAGE-A6 occurred in 29 (14.6%) and 52 (26%) patients whereas 170 (85.4%) and 147 (74%) patients were negative, respectively (Table 1). Metastasis and recurrence for tissues stained with MAGE-A11 occurred in 33 (15.5%) and 57 (27%) patients whereas 180 (84.5%) and 156 (73%) patients were negative, respectively (Table 2).

During the follow-up period, cancer-related death and the other cause of death were documented in 40 (78.4%) and 11 patients (21.6%) for MAGE-A6 expression and 42 (79.2%) and 11 patients (20.8%) for MAGE-A11, respectively. The mean and median follow-up durations were 77.34 (SD = 23.43) and 84 months for MAGE-A6 expression and 77.69 (SD = 23.87) and 84 months for MAGE-A11 expression, respectively; with a range of 1–99 months.

Kaplan–Meier analysis (with log-rank test) was used to investigate the association between MAGE-A6 and MAGE-A11 expressions with disease-specific survival (DSS) and progression-free survival (PFS). Based on the H-score described in the method section, nuclear and cytoplasmic expressions of MAGE-A6 and MAGE-A11 were divided into low, moderate or intermediate, and high expressions.

**Figure 4:**
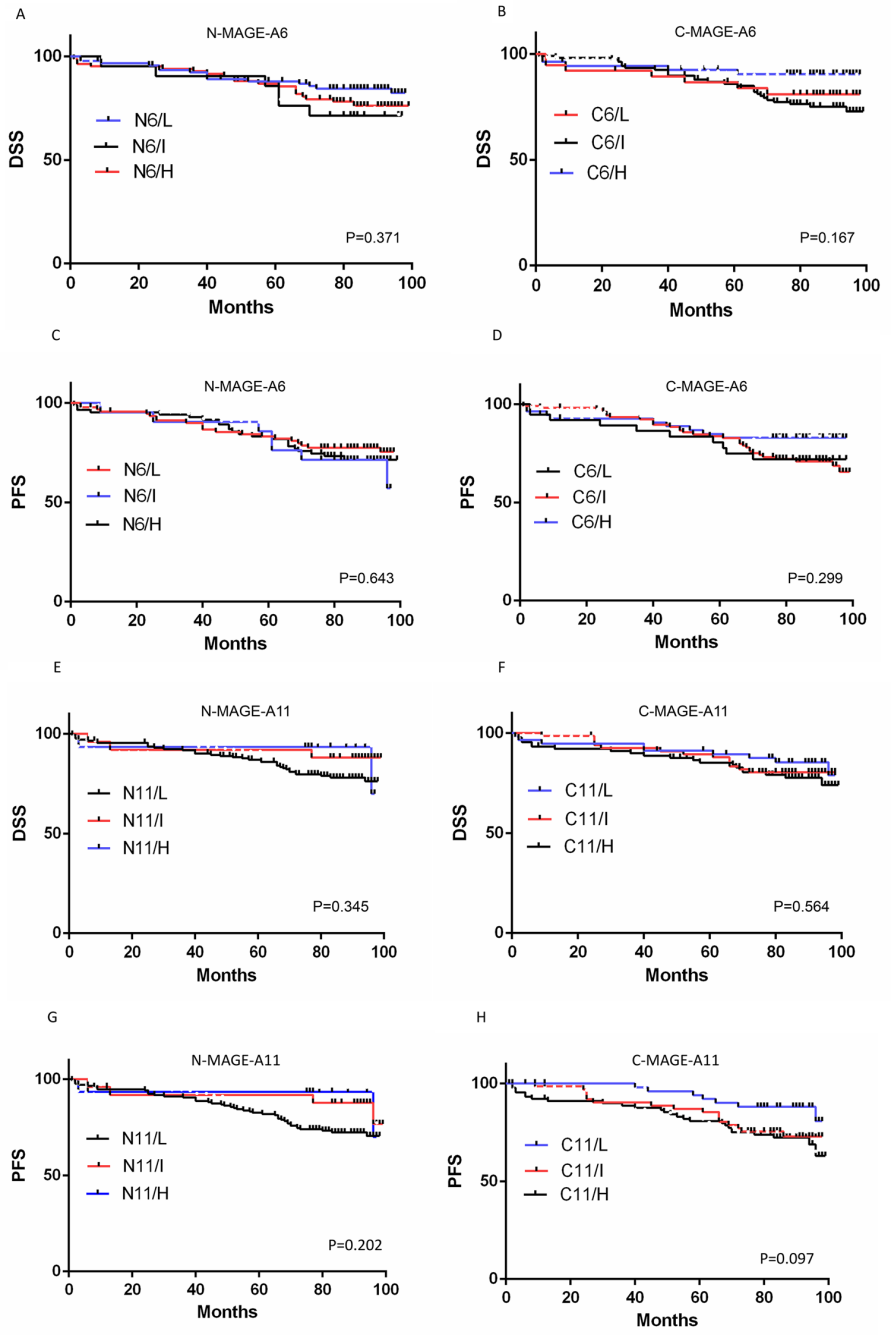
Survival analysis for MAGE-A6 expression **(A-D)** and MAGE-A11 **(E–H)** in BC patients (Kaplan–Meier analysis). The number of patients in the analyzed groups is as follows: For DSS in N6/L group: 93 (censored (C) = 78 and death (D) = 15) , N6/I : 21 (C = 15 and D = 6), N6/H : 85 (C = 66 and D = 19), C6/L: 38 (C = 31 and D = 7), C6/I : 107 (C = 80 and D = 27), and C6/H : 54 (C = 48 and D = 6). For PFS in N6/L group: 93 (C = 72 and D = 21), N6/I : 21 (C = 14 and D = 7), N6/H : 85 (C = 62 and D = 23), C6/L: 38 (C = 28 and D = 10), C6/I : 107 (C = 75 and D = 32), and C6/H : 54 (C = 45 and D = 9). For DSS in N11/L group: 172 (C = 135 and D = 37), N11/I: 25 (C = 22 and D = 3), N11/H :16 (C = 14 and D = 2), C11/L: 57 (C = 48 and D = 9), C11/I: 67 (C = 54 and D = 13), and C11/H : 89 (C = 69 and D = 20). For PFS in N11/L group: 172 (C = 125 and D = 47), N11/I : 25 (C = 21 and D = 4), N11/H : 16 (C = 14 and D = 2), C11/L:57 (C = 45 and D = 12), C11/I : 67 (C = 52 and D = 15), and C11/H : 89 (C = 63 and D = 26). C: cytoplasm, C6: cytoplasmic expression of MAGE-A6, C11: cytoplasmic expression of MAGE-A11, DSS: disease-specific survival, H: high expression, I: intermediate expression L: low expression, N: nuclear, N6: nuclear expression of MAGE-A6, N11: nuclear expression of MAGE-A11, P: p-value, PFS: progression free-survival. Charts were drawn by Prism version 8.3.0 software (Graph Pad Inc., San Diego, CA, USA). https://www.graphpad.com/support/faq/prism-830-release-notes/.

There was no significant association between nuclear and cytoplasmic expression of MAGE-A6 and MAGE-A11 with DSS and PFS of patients (for nuclear expression; P = 0.371, P = 0.643 (MAGE-A6), P = 0.345, P = 0.202 (MAGE-A11) and for cytoplasmic expression; P = 0.167, P = 0.299 (MAGE-A6), P = 0.564, P = 0.097 (MAGE-A11), respectively) (Fig. 4A–H).

The mean DSS rates for patients with low, intermediate, and high nuclear expressions of MAGE-A6 were 88.4 (SD = 2.49), 82.76 (SD = 5.55), and 86.8 (SD = 2.77) months, respectively. Low, intermediate, and high cytoplasmic expressions were also 85.67 (SD = 4.54), 86.44 (SD = 2.34), and 90.09 (SD = 3.22) months, respectively. Low, intermediate, and high nuclear expressions of MAGE-A11 were 86.14 (SD = 1.94), 90.96 (SD = 4.88), and 90.93 (SD = 5.67) months, respectively. Low, intermediate, and high cytoplasmic expression were also 89.34 (SD = 3.12), 87.92 (SD = 2.75), and 85.66 (SD = 2.99) months, respectively.

The mean PFS rates for patients with low, intermediate, and high nuclear expressions of MAGE-A6 were 84.58 (SD = 2.83), 82.61 (SD = 5.53), and 84.49 (SD = 2.89) months, respectively. Low, intermediate, and high cytoplasmic expressions were also 81.12 (SD = 4.96), 84.61 (SD = 2.43), and 86.51 (SD = 3.7) months, respectively. Low, intermediate, and high nuclear expressions of MAGE-A11 were 83.04 (SD = 2.09), 90.44 (SD = 4.96), and 90.93 (SD = 5.67) months, respectively. Low, intermediate, and high cytoplasmic expressions were also 86.82 (SD = 3.33), 85.57 (SD = 3.08), and 82.63 (SD = 3.15) months, respectively.

### Combined analysis of MAGE-A6/MAGE-A11 expression

To classify MAGE-A6 and MAGE-A11 phenotypes, the mean of H-score was evaluated as the cut-off point (for nuclear and cytoplasmic MAGE-A6, H-score = 157 and 197, respectively, for nuclear and cytoplasmic MAGE-A11, H-score = 57 and 197, respectively). Therefore, the expression of MAGE-A6 and MAGE-A11 phenotypes were classified into 4 subgroups; among 158 BC cases of cytoplasmic expression, 23 (14.6%) showed MAGE-A6^Low (l)^/MAGE-A11^l^ phenotype,38 (24.1%) MAGE-A6^high (h)^/MAGE-A11^l^, 22 (13.9%) MAGE-A6 ^l^/ MAGE-A11 ^h^ and 75(47.5%) MAGE-A6^h^/MAGE-A11^h^. For nuclear expression, 46 (31%) showed MAGE-A6^l^/MAGE-A11^l^ phenotype,67 (42.4%) MAGE-A6^h^/MAGE-A11^l^, 21 (13.3%) MAGE-A6^l^/ MAGE-A11^h^ and 21(13.3%) MAGE-A6^h^/MAGE-A11^h^.

The Pearson's chi-square analysis was used to examine the correlation between expression of MAGE-A6/MAGE-A11 phenotypes and clinicopathological parameters. Along with significant correlation of MAGE-A6 and MAGE-A11 with some clinicopathological parameters described above, significant differences were also found between both nuclear and cytoplasmic MAGE-A6/MAGE-A11 phenotypes with tumor size (P = 0.007, P = 0.043, respectively), different histological grades, PT stage, LP involvement, L/M involvement (all of the p-values for both phenotypes was P < 0.0001) (Tables 4 and 5). Pearson’s χ2 analysis showed that there was a direct and positive relationship between the mentioned parameters with MAGE-A6/MAGE-A11 phenotypes. As tumor size, histological grade, PT stage, tumor invasion to Laminia propia and L/M increased, simultaneous expression of MAGE-A6/MAGE-A11 increased.

**Table 4:** Association between nuclear MAGE-A6 (A6)/MAGE-A11 (A11) phenotypes and clinicopathological parameters in BC cases (P‐value, Pearson's chi‐square test).

Tumour characteristic	Phenotypes of Nuclear MAGE-A6/MAGE-A11 expression, N (%)				
	A6^l^/A11^l^	A6^h^/A11^l^	A6^l^/A11^h^	A6^h^/A11^h^	P-value
**Median age** Years					
≤ 65	28 (57.1)	24 (35.8)	11 (52.4)	10 (47.6)	0.133
> 65	21 (42.9)	43 (64.2)	10 (47.6)	11 (52.4)	
**Gender**					
Male	37 (75.5)	52 (77.6)	16 (76.2)	18 (85.7)	0.815
Female	12 (24.5)	15 (22.4)	5 (23.8)	3 (14.3)	
**Mean tumor size (cm)**					**0.007**
≤ 2.5	37 (75.5)	41 (61.2)	15 (28.6)	7 (33.3)	
> 2.5	12 (24.5)	26 (38.8)	6 (28.6)	14 (66.7)	
**Histological grade**					** < 0.0001**
Low	35 (71.4)	11 (16.4)	13 (61.9)	3 (14.3)	
High	14 (28.6)	56 (83.6)	8 (38.1)	18 (85.7)	
**pT stage**					
pTa	30 (61.2)	16 (24)	11 (52.4)	3 (14.3)	** < 0.0001**
pT1	13 (26.5)	36 (53.6)	8 (38.1)	13 (61.9)	
pT2	6 (12.2)	15 (22.4)	2 (9.5)	5 (23.8)	
pT3	0 (0)	0 (0)	0 (0)	0 (0)	
pT4	0 (0)	0 (0)	0 (0)	0 (0)	
**Lamina propria involvement**					** < 0.0001**
Involved	19 (38.8)	51 (76.1)	10 (47.5)	18 (85.7)	
None	30 (61.2)	16 (23.9)	11 (52.4)	3 (14.3)	
**Muscularis invasion**					0.319
Involved	6 (12.2)	15 (22.4)	2 (9.5)	5 (23.8)	
None	43 (87.8)	52 (77.6)	19 (90.5)	16 (76.2)	
**Lamina propria/muscularis involvement (L/M)**					** < 0.0001**
L − /M-	30 (61.2)	16 (23.9)	11 (52.4)	3 (14.3)	
L + /M-	13 (26.5)	36 (53.7)	8 (38.1)	13 (61.9)	
L + /M +	6 (12.2)	15 (22.4)	2 (9.5)	5 (23.8)	
Recurrence					0.349
Present	13 (26.5)	23 (34.3)	6 (28.6)	3 (14.3)	
Absent	36 (73.5)	44 (65.7)	15 (71.4)	18 (85.7)	
**Distant metastasis**					0.352
Present	8 (16.3)	13 (19.4)	1 (4.8)	2 (9.5)	
Absent	41 (83.7)	54 (80.6)	20 (95.2)	19 (90.5)	

Bold numbers represent significant p-values.

h: high expression, l: low expression.

**Table 5:** Association between cytoplasmic MAGE-A6 (A6)/MAGE-A11 (A11) phenotypes and clinicopathological parameters in BC cases (P‐value, Pearson's chi‐square test).

Tumour characteristic	Phenotypes of cytoplasmic MAGE-A6/MAGE-A11 expression, N (%)				
	A6^l^/A11^l^	A6^h^/A11^l^	A6^l^/A11^h^	A6^h^/A11^h^	P-value
**Median age**					
**Years (range)**					
65 ≤	11 (47.8)	18 (47.4)	12 (54.5)	32 (42.7)	0.792
65 >	12 (52.2)	20 (52.6)	10 (45.5)	43 (57.3)	
**Gender**					
Male	18 (78.3)	30 (78.9)	15 (68.2)	60 (80)	0.7
Female	5 (21.7)	8 (21.1)	7 (31.8)	15 (20)	
**Tumor size (cm)**					
2.5 ≤ Mean	20 (87)	24 (63.2)	15 (68.2)	41 (54.7)	**0.043**
2.5 > Mean	30 (13)	14 (36.8)	7 (31.8)	34 (45.3)	
**Histological grade**					
Low	17 (73.9)	19 (50)	12 (54.5)	14 (18.7)	** < 0.0001**
High	6 (26.1)	19 (50)	10 (45.5)	61 (81.3)	
**pT stage**					
pTa	16 (69.6)	18 (47.4)	11 (50)	15 (20)	** < 0.0001**
pT1	5 (21.7)	11 (28.9)	9 (41)	45 (60)	
pT2	2 (8.7)	9 (23.7)	2 (9)	15 (20)	
pT3	0 (0)	0 (0)	0 (0)	0 (0)	
pT4	0 (0)	0 (0)	0 (0)	0 (0)	
**Lamina propria involvement**					
Involved	7 (30.4)	20 (52.6)	11 (50)	60 (80)	** < 0.0001**
None	16 (69.6)	18 (47.4)	11 (50)	15 (20)	
**Muscularis invasion**					0.308
Involved	2 (8.7)	9 (23.7)	2 (9.1)	15 (20)	
None	21 (91.3)	29 (76.3)	20 (90.9)	60 (80)	
**Lamina propria/muscularis involvement (L/M)**					** < 0.0001**
L − /M-	16 (69.6)	18 (47.4)	11 (50)	15 (20)	
L + /M-	5 (21.7)	11 (28.9)	9 (40.9)	40 (60)	
L + /M +	2 (8.7)	9 (23.7)	2 (9.1)	15 (20)	
**Recurrence**					
Present	4 (17.4)	10 (26.3)	9 (40.9)	22 (29.3)	0.366
Absent	19 (82.6)	28 (73.7)	13 (59.1)	53 (70.7)	
**Distant metastasis**					
Present	2 (8.7)	6 (15.8)	4 (18.2)	12 (16)	0.812
Absent	21 (91.3)	32 (84.2)	18 (81.8)	63n	

Bold numbers represent significant p-values.

h: high expression, l: low expression.

To know which phenotypes caused significant differences, one‐way ANOVA and Tukey s post hoc analysis were used to examine the correlation between expressions of MAGE-A6/MAGE-A11 phenotypes and clinicopathological parameters. As shown in Table 6, there was a significant correlation mainly between MAGE-A6^h^/MAGE-A11^h^ phenotype with other phenotypes for clinicopathological variables. These findings indicate the importance of the high expression of these two markers in tissue samples of patients with BC.

**Table 6:** Association between nuclear and cytoplasmic MAGE-A6 (A6)/MAGE-A11 (A11) phenotypes and clinicopathological parameters in BC cases (P‐value, one‐way ANOVA and Tukey s post hoc).

Tumor characteristic	Nuclear phenotypes	P value	Cytoplasmic phenotypes	P value
Tumor size (cm)	A6^l^/A11^l^ & A6^h^/A11^h^ A6^l^/A11^h^ & A6^h^/A11^h^	0.004 0.046	A6^l^/A11^l^ & A6^h^/A11^h^	0.02
Histological grade	A6^l^/A11^l^ & A6^h^/A11^l^ A6^l^/A11^l^ & A6^h^/A11^h^ A6^h^/A11^l^ & A6^l^/A11^h^ A6^l^/A11^h^& A6^h^/A11^h^	< 0.0001 < 0.0001 < 0.0001 0.002	A6^l^/A11^l^ & A6^h^/A11^h^ A6^h^/A11^l^ & A6^h^/A11^h^ A6^l^/A11^h^ & A6^h^/A11^h^	< 0.0001 0.003 0.006
pT stage	A6^l^/A11^l^ & A6^h^/A11^l^ A6^l^/A11^l^ & A6^h^/A11^h^	0.002 0.007	A6^l^/A11^l^ & A6^h^/A11^h^	0.002
Lamina propria	A6^l^/A11^l^ & A6^h^/A11^l^ A6^l^/A11^l^ & A6^h^/A11^h^ A6^l^/A11^h^ & A6^h^/A11^h^	< 0.0001 0.001 0.036	A6^l^/A11^l^ & A6^h^/A11^h^ A6^h^/A11^l^ & A6^h^/A11^h^ A6^l^/A11^h^ & A6^h^/A11^h^	< 0.0001 < 0.0001 0.036
Lamina propria/muscularis	A6^l^/A11^l^ & A6^h^/A11^l^ A6^l^/A11^l^ & A6^h^/A11^h^	0.001 0.002	A6^l^/A11^l^ & A6^h^/A11^h^ A6^h^/A11^l^ & A6^h^/A11^h^	< 0.0001 0.004

It should be noted that only p-value of phenotypes that were significant with clinicopathological parameters were shown.

**Figure 5:**
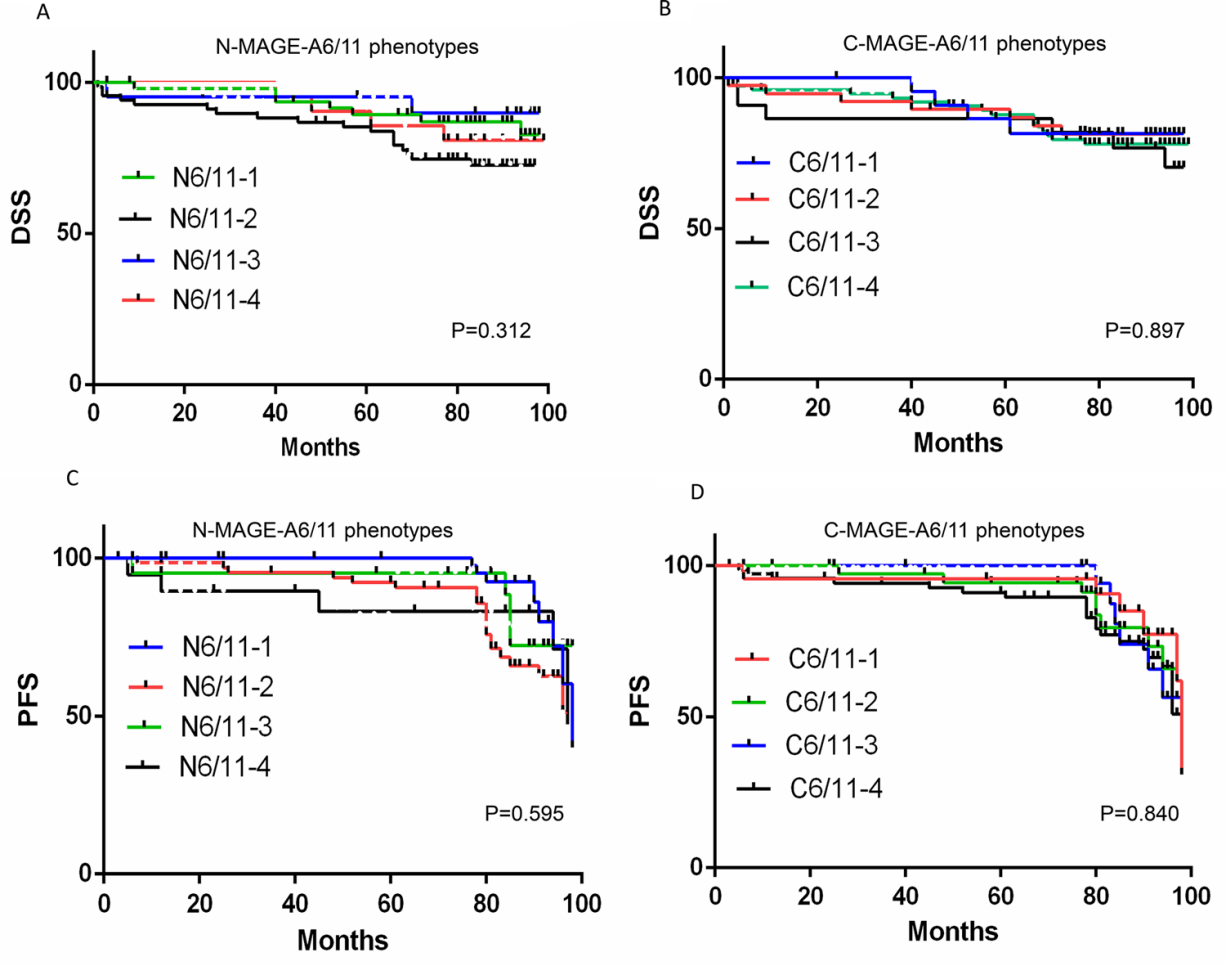
Survival analysis for nuclear and cytoplasmic MAGE-A6/MAGE-A11 phenotypes. (A-D; Kaplan–Meier analysis). The number of patients in the analyzed groups is as follows: For DSS in N6/11–1 phenotype 49 (censored (C) = 40 and death (D) = 9), N6/11–2: 67 (C = 49 and D = 18), N6/11–3: 21 (C = 19 and D = 2), N6/11–4: 21 (C = 17 and D = 4), C6/11–1: 23 (C = 19 and D = 4), and C6/11–2: 38 (C = 31 and D = 17), C6/11–3 : 22 (C = 16 and D = 6), and C6/11–4: 75 (C = 59 and D = 16). For PFS in N6/11–1 phenotype 49 (C = 37 and D = 12), N6/11–2: 67 (C = 46 and D = 21), N6/11–3: 21 (C = 17 and D = 14), N6/11–4: 21 (C = 16 and D = 5), C6/11–1: 23 (C = 17 and D = 6), and C6/11–2: 38 (C = 30 and D = 8), C6/11–3: 22 (C = 16 and D = 6), and C6/11–4: 75 (C = 53 and D = 22). C: cytoplasmic, C6/11: cytoplasmic expression of MAGE-A6 and MAGE-A11, DSS: disease-specific survival, N: nuclear, N6/11: nuclear expression of MAGE-A6 and MAGE-A11, P: p-value, PFS: progression-free survival, 1: MAGE-A6^low^/MAGE-A11^low^ phenotype, 2: MAGE-A6^low^/MAGE-A11^high^ phenotype, 3: MAGE-A6^low^/MAGE-A11^high^ phenotype, 4: MAGE-A6^high^/MAGE-A11^high^ phenotype. Charts were drawn by Prism version 8.3.0 software (Graph Pad Inc., San Diego, CA, USA). https://www.graphpad.com/support/faq/prism-830-release-notes/.

Consistent to survival analysis for expression of MAGE-A6 and MAGE-A11 separately, there was no significant association between nuclear and cytoplasmic MAGE-A6/MAGE-A11 phenotypes with DSS and PFS of patients (for nuclear phenotypes; P = 0.312, P = 0.595 and for cytoplasmic phenotypes; P = 0.897, P = 0.840, respectively) (Fig. 5 A–D).

The mean DSS rates for nuclear MAGE-A6 ^l^/MAGE-A11 ^l^, MAGE-A6 ^h^/MAGE-A11^l^, MAGE-A6^l^/MAGE-A11^h^, and MAGE-A6 ^h^/MAGE-A11 ^h^ phenotypes were 88.53 (SD = 3.28), 82.01 (SD = 3.46), 91.99 (SD = 4.57), and 90.9 (SD = 3.87) months and for cytoplasmic phenotypes 89.08 (SD = 4.08), 86.26 (SD = 4.08), 83.02 (SD = 6.76), and 87.7 (SD = 2.81), respectively. The mean PFS rates for nuclear MAGE-A6 ^l^/MAGE-A11 ^l^, MAGE-A6 ^h^/MAGE-A11^l^, MAGE-A6^l^/MAGE-A11^h^, and MAGE-A6 ^h^/MAGE-A11 ^h^ phenotypes were 84.68 (SD = 3.81), 80.08 (SD = 3.53), 85.96 (SD = 5.93), and 90.41 (SD = 3.85) months and for cytoplasmic phenotypes 83.33 (SD = 5.26), 84.74 (SD = 4.25), 82.88 (SD = 6.76), and 84.08 (SD = 3.08) months, respectively.

## Discussion

Despite recent progress in BC prognostic, we faced a wide range of failures in bladder patients' treatment^56^. Thus, novel and practical clinical prognostic markers are needed to be introduced for future BC decision-making in a clinical settlement. In this way, the bioinformatics analysis would be conducive to defining novel molecular markers. In the current study, we tend to identify the biomarkers that were potentially involved in the BC. Regarding this, we focused on GEO microarray analysis and some bioinformatics online software investigations of important genes. Besides, enrichment analysis determined the involved pathways and their molecular function. Among them, we selected the MAGE-A gene family that is expressed at a high frequency in various tumors. The expression of MAGE-A11 and MAGE-A6 genes were examined only in two studies that were performed by Duan et al.^57^ and Laiseca et al.^58^ based on coremine data (https://www.coremine.com/) to clarify MAGE proteins collaboration in tumorigenesis and the potential importance of their detection to prognosis purposes. Therefore, for the first time, to validate the MAGE-A11 and MAGE-A6 proteins as a prognostic marker for BC was investigated in a well-characterized series of BC tissues specimens.

In this study, we evaluated immunohistochemical analysis for the expression pattern of MAGE-A6 and MAGE-A11 in 199 and 213 BC samples, respectively. The expression patterns were analyzed with the clinicopathological data of the patients with BC including age, gender, tumor size, histological grade, PT stage, LP involvement, muscle invasion, L/M involvement, recurrence, and distance metastasis. Survival analysis was also evaluated to find the vitality of the MAGE-A6 and MAGE-A11 as potential prognostic factors.

MAGE-A11 contributes to the AR signaling pathway in prostate cancer cells^23^. It binds directly to the AR, promoting transcriptional through direct binding to the AR FXXLF motif region^23^. Previous studies have demonstrated that AR activation generally associates with the promotion of the growth and development of BC^59–61^. Such that AR deletion in AR-positive bladder cell lines using siRNA led to a significantly decreased cell proliferation, cyclin-D1, Bcl-x(L) as well as migration, metastasis-related matrix metallopeptidase (MMP)-9 compared to control^62^. Moreover, it has been shown that androgen-mediated AR signals are correlated with the tumor development and progression of cancer, which may obviously justify some sex-specific differences in BC^59^. Database search confirmed that MAGE-A6 and MAGE-A11 are co-expressed in samples of human prostate cancer. The interaction between these two markers in cancer progression is clearly elucidated in an experimental study^27^.

In the current study, MAGE-A6 and MAGE-A11 were detected in cancer cells either in the cytoplasm or the combined pattern of staining as the nucleus & cytoplasm pattern. Our staining pattern was consistent with findings of Jia Sh et al. for MAGE-A11 expression in head and neck Squamous cell carcinoma^24^. Endo et al. also evaluate MAGE-A6 in gastric cancer by immunohistochemistry, but there is no explanation for the pattern of MAGE-A6 expression in this study^28^. Our immunohistochemical staining showed different expression patterns, from negative to strong staining, so that there was a differentiation between low grades and high grades of BC for both nuclear and cytoplasmic MAGE-A6 expressions as well as cytoplasmic MAGE-A11 expression. Simultaneous nuclear and cytoplasmic expression of other members of the MAGE-A family including MAGE-A2 and MAGE-A3 in patients with prostate cancer revealed a significant correlation with clinic-pathological outcomes and recurrence-free survival^13,63^. Similarly, the expression pattern of MAGE-A6 and MAGE-A11 in BC and their correlation with clinic-pathological findings indicate the importance of expression of some members of the MAGE-A family in the diagnosis and prognosis of cancer patients. It was suggested that the pattern of subcellular expression of these antigens may indicate a difference in their biological activity.

A high level of MAGE-A11 protein was found in castration-recurrent prostate cancer. Increased MAGE-A11 levels participate in AR transactivation and the growth and progression of prostate cancer^23^. On the side, Endo et al. revealed that a high level of MAGE-A6 is associated with a worse prognosis in patients with gastric cancer. Its expression level in primary lesions predicted the possibility of disease recurrence. MAGE-A6 mRNA levels were higher in gastric cancer tissues in comparison with normal tissues. A positive correlation was also found between the mRNA of MAGE-A6 and matrix metallopeptidase 9 mRNA^28^. In a systematic literature search, MAGE-A6 was also significant in thymoma, esophageal adenocarcinoma, and kidney renal papillary cell carcinoma, while MAGE-A11 was in pheochromocytoma and paraganglioma^64^. Increased MAGE-A11 was also an independent prognostic factor for the overall survival in patients with head and neck squamous cell carcinomas^24^.

In this study, a strong significant direct correlation was observed between the expression of nuclear and cytoplasmic MAGE-A6 as well as cytoplasmic MAGE-A11 with histological grade, PT stage, LP involvement, and L/M involvement, so that with increasing grade, stage, and tumor invasion into LP and L/M, the expression of these two markers increased. Consistently, when the pathological grade of patients was analyzed using Mann–Whitney U test, a direct correlation was also found between both nuclear and cytoplasmic expressions of MAGE-A6 and cytoplasmic expression of MAGE-A11. These findings indicate the importance of the high expression of these markers in the progression of BC. Moreover, the significance of cytoplasmic expression of the MAGE-A11 was valuable with clinicopathological features that could indicate the activity of this marker in the cytoplasm of cancer cells, while both nuclear and cytoplasmic expression of MAGE-A6 was valuable with clinicopathological features. Although the exact role of MAGE-A6 is unknown, these findings indicate the importance of the expression of this marker as well as its possible function in the nucleus and cytoplasm of BC cells.

In our study, survival analysis was performed and the association of MAGE-A6 and MAGE-A11 expressions with DSS and PFS was determined. Univariate analysis indicated that there was no association for both MAGE-A6 and MAGE-A11 expressions with DSS and PFS. Although the PFS was longer in patients with cytoplasmic poor expression of the MAGE-A11; however, no significant association was observed for their expression. This may be due to the higher number of patients in the censored group than the death group, such that the number of cancer-related deaths was low during the follow-up period; if the follow-up period extended, the number of deaths may increase relative to patient survival.

It has been shown that the MHD domain of MAGE-A6 enhances AR activation through MAGE-A11 and is critical for MAGE-A11 interaction and AR regulation. When MAGE-A6 is co-expressed, a lower degree of MAGE-A11 was ubiquitinylation suggesting that it could protect MAGE-A11 from proteasome-dependent degradation proteins^27^. However, no effect on the dynamics of AR translocation to the nucleus was observed upon MAGE-A6 expression^27^. Therefore, due to the interaction of these two markers in the activation of AR and the spread of malignant progression, we attempted to identify different phenotypes with regard to MAGE-A6 and MAGE-A11 expressions in BC tissues. We compared the significance of MAGE-A6 and MAGE-A11 co‐expression in clinical samples in BC. For cytoplasmic expression, the highest percentage among bladder samples was allocated to the MAGE-A6^h^/MAGE-A11^h^ phenotype with 47.5% cases. In addition, statistical analysis showed that there is a bivariate correlation between cytoplasmic MAGE-A6 and MAGE-A11 expressions in bladder samples. It is suggested that these markers affect the expression of each other in the cytoplasm of cancer cells. According to the analyzes described for each marker separately above, combined analyzes also showed a significant association of various phenotypes of the MAGE-A6/MAGE-A11 with clinicopathological parameters including tumor size, histological grade, PT stage, LP involvement, and L/M involvement for both nuclear and cytoplasmic expression. The major phenotype that caused significant differences in clinicopathological parameters was the MAGE-A6^h^/MAGE-A11^h^. In other words, when the expression of both markers increases simultaneously, the rate of disease progression based on clinicopathological parameters was significant that indicate the importance of high expression of these two markers in tissue samples of patients with BC. These immunopathological data were in line with the previous in vitro study that showed the MAGE-A6 and MAGE-A11 form a protein complex resulting in the stabilization of MAGE-A11 and consequently the enhancement of AR activity^27^.

On the other hand, given that the importance of the complex formation of MAGE-A6/MAGE-A11 in activating AR, however, our immunohistochemical study failed to reveal significant sex-related differences for both MAGE-A6 and MAGE-A11 expressions in male versus female samples. This finding is consistent with previous findings that showed no significant sex-related differences in AR expression in male versus female tissues in patients^59^. However, in order to determine the exact mechanism of action of the MAGE-A6 and MAGE-A11 in BC cells, future studies are needed to answer the question of whether these two markers really function in the progression of BC through ARs?

On the other hand, in recent years, immunotherapy has played a major role in the treatment of cancer patients. To establish immunotherapy or vaccination against tumor immunogenic antigens and eventually prolonged survival of patients, expression of these antigens should first be examined in a well-characterized series of cancer tissue specimens, which this experiment currently carried out for MAGE-A6 and MAGE-11 expressions in BC. Poor presentation of MAGE-As in normal tissues, increased expression of theses antigens in malignant tissues, and their high immunogenicity has directed experiments into utilizing them as targets for cancer immunotherapies^19^. We found that MAGE-A6 and MAGE-A11 are highly expressed in BC tissues with high-grade malignancy, therefor it is promising interest to establish the BC immunotherapy for restricting tumor cells through activating specific CD8^+^ T cells (cytotoxic T lymphocyte; CTL) against tumor cells expressing MAGE-A6 or MAGE-A11.

Taken together, the strength of the association between clinicopathological parameters and immunoreactive MAGE-A6 and MAGE-A11 scoring as well as MAGE-A6/MAGE-A11 co-expression can promote the potentials of these markers for diagnosis and progression of BC. It is suggested that the increased MAGE-A6 expression can influence the MAGE-A11 expression in BC tissues; however, there are still some ambiguities in the clinical significance of MAGE-A6 and MAGE-A11 expression levels that require future studies.

## Material and methods

### Bioinformatics approach

GEO database was searched (https://www.ncbi.nlm.nih.gov/geo/) to identify BC studies^65^. Then, the Venn diagram analysis was performed to find a common significant differential in the output by Venny (https://bioinfogp.cnb.csic.es/tools/venny/index2.0.2.html)^66^. Common genes of these three types of research were selected for subsequent analysis on Enrichr (amp.pharm.mssm.edu/Enrichr/) based on GO (http://geneontology.org/)^67^. GO analysis consist of cellular component (CC), biological process (BP), and molecular function (MF). Besides, pathways including Reactome (https://reactome.org/) were applied^68^. Additionally, we tried to use a PPI network with more related genes connections^69^. Next, we applied the cBioPortal (https://www.cbioportal.org/) which is a tool for collecting next-generation sequencing data from the TCGA and the international cancer genome consortium (ICGC) that is a repository of user-friendly cancer genomics datasets^70,71^. Furthermore, the online database GEPIA, for analyzing the RNA sequencing expression data and prognostic value were used. All samples in the GEPIA database were derived from the genotype-tissue expression (GTEx) and TCGA projects (http://gepia.cancerpku.cn/index.html)^72^. Also, the protein expression level of these selected genes was considered in BC on the UALCAN database (http://ualcan.path.uab.edu/) which provides protein expression analysis options using data from TCGA^73^. Additionally, in the Cytoscape (https://cytoscape.org/) our genes were investigated in BC samples^74^.

Additionally, we applied MAGE-A6 and MAGEA-11 in the EBI’s Expression Atlas website (https://www.ebi.ac.uk/gxa/home) in order to confirm these invaluable targets expression in bladder carcinoma/ bladder tumor cell lines^75^.

### Patients’ characteristics and tissue collection

A total of 250 formalin-fixed, paraffin-embedded (FFPE) specimens were collected from BC patients after transurethral resection of bladder tumor (TURB) with no preoperative chemotherapy or radiotherapy in the Hasheminejad Urology-Nephrology Hospital, Iran, between 2008 to 2011. Of this collection, 51 specimens during MAGE-A6 staining and 37 specimens during MAGE-A11 staining were excluded from the study due to technical problems in tissue processing, leaving a total of 199 and 213 cases for the final evaluation, respectively. Medical documents and hematoxylin and eosin (H&E) stained slides were reviewed to collect the following pathological and clinical characteristics: age, gender, tumor size (maximum tumor diameter), histological grade, pT stage, lamina propria^60^ involvement, muscularis invasion, L/M involvement, distant metastasis, and tumor recurrence. Furthermore, 11 adjacent normal tissue samples were used to evaluate the expression of MAGE-6 or MAGE-11 compared to cancerous tissues. The cut-off size and the pT stage of cancers were defined based on the American joint committee on cancer/international union against cancer (AJCC/UICC) and pTNM classification, respectively^76–78^. In addition, the patient’s survival data, including DSS and PFS, was recorded. DSS was calculated from the time of surgery to the time of death related to the patient’s tumor and PFS was considered as an interval between the primary surgery and the last follow-up visit if the case exhibited no sign of disease, recurrence, or distant metastasis.

### Tissue microarray construction

BC tissue microarrays were constructed as described previously^79^. Briefly, H&E slides of all specimens were evaluated by an experienced pathologist (M.A) to select and mark out three suitable regions of cancer in each block. Then, selected spots of each primary block were punched out with a diameter of 0.6 mm and transferred into the TMA recipient paraffin blocks through a precision arraying instrument (ALPHELYS, Plaisir, France). In the present research, due to the issue of tumor heterogeneity, three cores were constructed for each specimen and, then, scored separately to obtain better results^80^. The mean of the three cores was considered as the final score for each tissue specimen.

### Immunohistochemistry staining

The expression of MAGE-6 and MAGE-11 was immunohistochemically assessed through our laboratory protocol^81^. Briefly, all TMA sections were deparaffinized in xylene, and then rehydrated through immersion in reducing grades of ethanol. Subsequently, methanol containing 0.3% hydrogen peroxide (H2O2) was used to suppress the activity of endogenous peroxidase. After washing the tissue sections three times in Tris Buffered Saline (TBS), the slides were autoclaved for 10 min in sodium citrate buffer (pH 6.0) to retrieve the antigens. The slides were treated overnight at 4 °C with the following antibody dilutions as the optimal dilution for subsequent use: anti-MAGE-6 antibody (Sigma Aldrich, USA) using a 1:100 dilution, and anti-MAGE-11 antibody (Abcam, Inc., Cambridge, MA) using a 1:150 dilution. The next day, TMA slides were washed with TBS and, then, incubated with the secondary antibody, EnVision™ + /HRP, DualLink Rabbit/Mouse (Dako, Denmark) for 1 h at room temperature. Subsequently, visualization of the antigen was achieved through 3, 3′-diaminobenzidine (DAB) chromogen substrate for 10 min at room temperature followed by counterstaining with hematoxylin (Dako). Finally, sections were dehydrated with graded alcohol, cleared in xylene (Dako), and mounted for evaluation. Moreover, a non‐reactive primary antibody was used instead of the primary MAGE‐A6 or MAGE-A11 antibody as the negative controls. Liver and prostate tissues were used as a positive control for MAGE-A6 and MAGE-A11, respectively.

### Immunostaining evaluation and scoring system

The immunohistochemical staining of tissue slides was scored through a semi-quantitative scoring system by a professional pathologist (M.A) who was blinded to pathological and clinical data associated with each sample.

The intensity of immunostaining was scored on a 4-point scale as follows: 0 (negative), 1 (weak), 2 (intermediate), and 3 (strong or high). The percentage of positive cells was valued from 0 to 100%. The overall score was achieved through H-score (histochemical score) for each patient by multiplying the intensity score (0–3) to the percentage of the positive cells (0–100%), obtaining a range from 0 to 300. In this study, H-score was categorized into three groups: 0–100 as group 1 (low expression), 101–200 as group 2 (intermediate expression), and 201–300 as group 3 (high expression).

### Statistical analysis

Statistical analyses were carried out through the SPSS statistical software package version 25 (SPSS, Chicago, IL, USA). The association and correlation between MAGE-A6 or MAGE-A11 expression and clinicopathological characteristics were analyzed using Pearson’s *χ*2, *R* tests, and One-way ANOVA. The pairwise comparisons across the groups were performed through Mann–Whitney *U* test. Survival analysis was estimated through the Kaplan–Meier method and the estimated curves across the groups were compared using the log-rank test. A p-value of < 0.05 was regarded as statistically significant. Charts were drawn through Prism version 8.3.0 software (Graph Pad Inc., San Diego, CA, USA) and SPSS graphs.

### Ethical approval

This study was confirmed by the Human Research Ethics Committee of Iran University of Medical Sciences in Iran (Ref No: IR.IUMS.REC.1398.682) and signed informed consent was obtained from all patients participating in this research. All procedures were performed according to the 1964 Helsinki Declaration and its later amendments.

## Supplementary Information

Supplementary Figure 1.

Supplementary Figure 2.

Supplementary Table 1.
