# Publisher Correction: Co-expression of cancer-testis antigens of MAGE-A6 and MAGE-A11 is associated with tumor aggressiveness in patients with bladder cancer

**DOI:** 10.1038/s41598-022-06282-9

**Published:** 2022-02-07

**Authors:** Monireh Mohsenzadegan, Mahdieh Razmi, Somayeh Vafaei, Maryam Abolhasani, Zahra Madjd, Leili Saeednejad Zanjani, Laleh Sharifi

**Affiliations:** 1 Department of Medical Laboratory Science, Faculty of Allied Medical Sciences, Iran University of Medical Sciences (IUMS), Hemmat Highway, Tehran, Iran; 2 Oncopathology Research Center, Iran University of Medical Sciences (IUMS), Hemmat Street (Highway), Next To Milad Tower, 1449614535 Tehran, Iran; 3 Department of Molecular Medicine, Faculty of Advanced Technologies in Medicine, Iran University of Medical Sciences, Tehran, Iran; 4 Department of Pathology, School of Medicine, Iran University of Medical Sciences, Tehran, Iran; 5 Hasheminejad Kidney Center, Iran University of Medical Sciences, (IUMS), Tehran, Iran; 6 Uro‑Oncology Research Center, Tehran University of Medical Sciences (TUMS), Tehran, Iran

Correction to: *Scientific Reports* https://doi.org/10.1038/s41598-021-04510-2, published online 12 January 2022

The original version of this Article contained errors.

In Figure 2, panels (G), (H) and (I) were omitted. The original Figure 2 and accompanying legend appear below.

**Figure 2:**
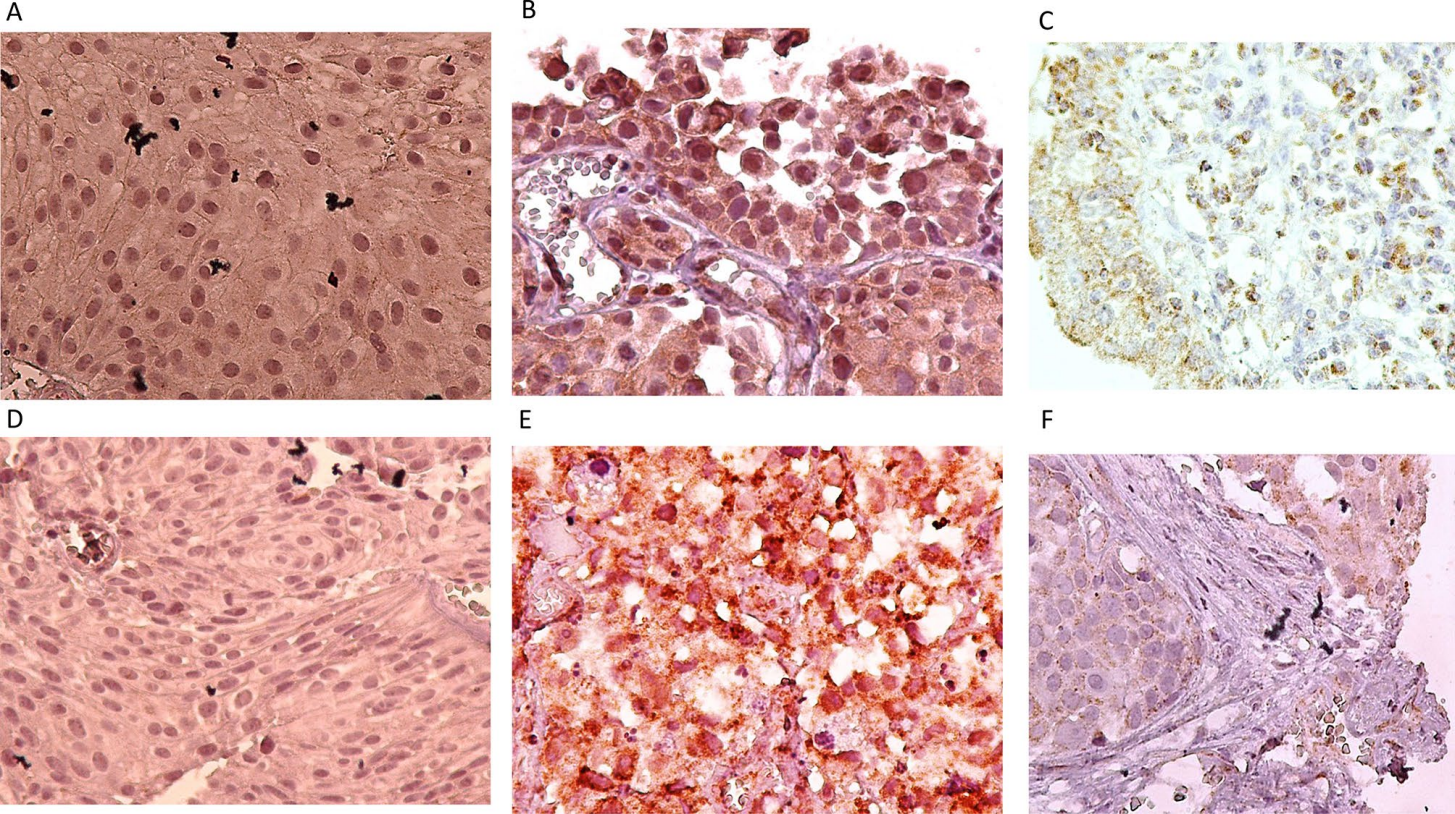
Staining pattern of MAGE-A6 expression (**A**–**C**) and MAGE-A11 expression (**D**–**F**) in bladder tissues. (**A**) Intermediate staining for both nuclear and cytoplasmic expressions in low-grade BC (pTa stage), (**B**) Strong staining for nuclear expression and intermediate staining for cytoplasmic expression in high-grade BC (pT1 stage), (**C**) MAGE-A6 expression in adjacent normal tissue, (**D**) Weak staining for both nuclear and cytoplasmic expressions in low-grade BC (pTa stage), (**E**) Strong staining for both nuclear and cytoplasmic expressions in high-grade BC (pT2 stage), (**F**) MAGE-A11 expression in adjacent non-tumoral tissue, (**G**) MAGE-A6 expression in liver tissue as a positive control, (**H**) MAGE-A11 expression in prostate tissue as a positive control, and (**I**) Staining of bladder tissue with a nonreactive antibody (anti-CD11b antibody, negative control). All images were taken at 400× magnification.

Similarly, in Figure 4, panels (C)–(H) and in Figure 5, panels (C) and (D) were omitted. The original Figures 4 and 5 and their accompanying legends appear below.

**Figure 4:**
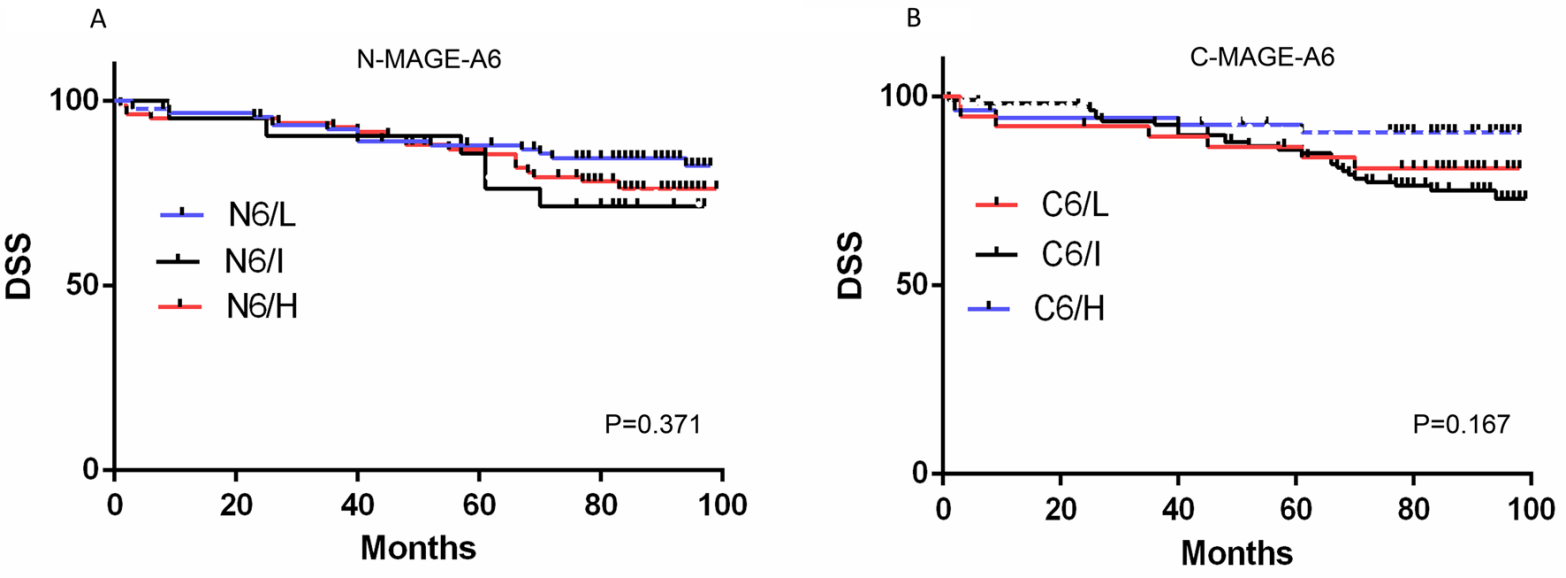
Survival analysis for MAGE-A6 expression (**A**–**D**) and MAGE-A11 (**E**–**H**) in BC patients (Kaplan–Meier analysis). The number of patients in the analyzed groups is as follows: For DSS in N6/L group: 93 (censored (C) = 78 and death (D) = 15) , N6/I: 21 (C = 15 and D = 6), N6/H: 85 (C = 66 and D = 19), C6/L: 38 (C = 31 and D = 7), C6/I: 107 (C = 80 and D = 27), and C6/H: 54 (C = 48 and D = 6). For PFS in N6/L group: 93 (C = 72 and D = 21), N6/I: 21 (C = 14 and D = 7), N6/H: 85 (C = 62 and D = 23), C6/L: 38 (C = 28 and D = 10), C6/I: 107 (C = 75 and D = 32), and C6/H: 54 (C = 45 and D = 9). For DSS in N11/L group: 172 (C = 135 and D = 37), N11/I: 25 (C = 22 and D = 3), N11/H:16 (C = 14 and D = 2), C11/L: 57 (C = 48 and D = 9), C11/I: 67 (C = 54 and D = 13), and C11/H: 89 (C = 69 and D = 20). For PFS in N11/L group: 172 (C = 125 and D = 47), N11/I: 25 (C = 21 and D = 4), N11/H: 16 (C = 14 and D = 2), C11/L:57 (C = 45 and D = 12), C11/I: 67 (C = 52 and D = 15), and C11/H: 89 (C = 63 and D = 26). C: cytoplasm, C6: cytoplasmic expression of MAGE-A6, C11: cytoplasmic expression of MAGE-A11, DSS: disease-specific survival, H: high expression, I: intermediate expression L: low expression, N: nuclear, N6: nuclear expression of MAGE-A6, N11: nuclear expression of MAGE-A11, P: *p* value, PFS: progression free-survival. Charts were drawn by Prism version 8.3.0 software (Graph Pad Inc., San Diego, CA, USA). https://www.graphpad.com/support/faq/prism-830-release-notes/.

**Figure 5:**
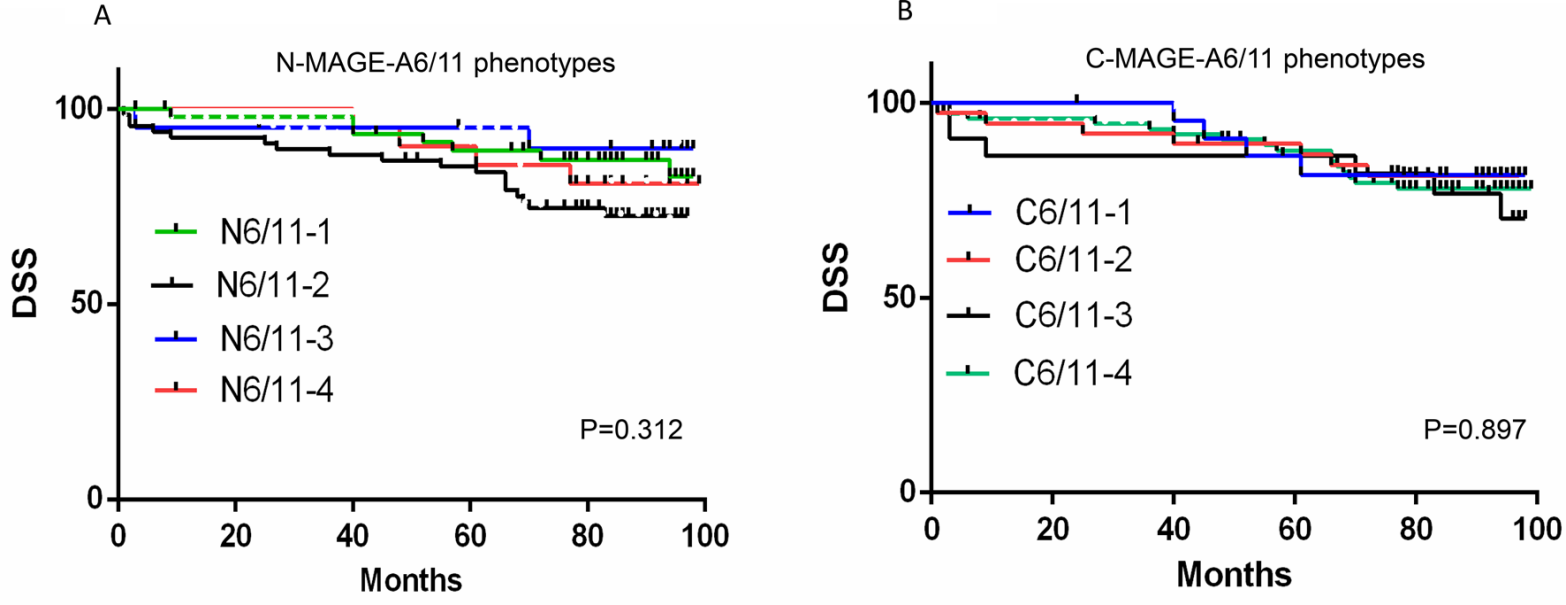
Survival analysis for nuclear and cytoplasmic MAGE-A6/MAGE-A11 phenotypes. (**A**–**D**; Kaplan–Meier analysis). The number of patients in the analyzed groups is as follows: For DSS in N6/11–1 phenotype 49 (censored (**C**) = 40 and death (**D **) = 9), N6/11–2: 67 (C = 49 and D = 18), N6/11–3: 21 (C = 19 and D = 2), N6/11–4: 21 (C = 17 and D = 4), C6/11–1: 23 (C = 19 and D = 4), and C6/11–2: 38 (C = 31 and D = 17), C6/11–3: 22 (C = 16 and D = 6) , and C6/11–4: 75 (C = 59 and D = 16) . For PFS in N6/11–1 phenotype 49 (C = 37 and D = 12), N6/11–2: 67 (C = 46 and D = 21), N6/11–3: 21 (C = 17 and D = 14), N6/11–4: 21 (C = 16 and D = 5), C6/11–1: 23 (C = 17 and D = 6), and C6/11–2: 38 (C = 30 and D = 8), C6/11–3: 22 (C = 16 and D = 6) , and C6/11–4: 75 (C = 53 and D = 22). C: cytoplasmic, C6/11: cytoplasmic expression of MAGE-A6 and MAGE-A11, DSS: disease-specific survival, N: nuclear, N6/11: nuclear expression of MAGE-A6 and MAGE-A11, P: *p* value, PFS: progression-free survival, 1: MAGE-A6^low^/MAGE-A11^low^ phenotype, 2: MAGE-A6^high^/MAGE-A11^low^ phenotype, 3: MAGE-A6^low^/MAGE-A11^high^ phenotype, 4: MAGE-A6^high^/MAGE-A11^high^ phenotype. Charts were drawn by Prism version 8.3.0 software (Graph Pad Inc., San Diego, CA, USA) https://www.graphpad.com/support/faq/prism-830-release-notes/.

Additionally, in Table 1, values in columns “Total samples N (%)”, “Nuclear expression of MAGE-A11” and “Cytoplasmic expression of MAGE-A11” were omitted. The original Table 1 and accompanying legend appear below.

**Table 1:** Association between MAGE-A6 expressions (staining intensity and H-score) and clinic-pathological parameters of BC cases (*p* value, Pearson’s chi-square test).

Patients and tumor characteristics	Total samples N (%)	Nuclear expression of MAGE-A11		Cytoplasmic expression of MAGE-A11	
		Staining Intensity	H-score	Staining Intensity	H-score
**Median age**					
**Years**					
≤ 67	108 (51)	0.079		0.638	
> 67	105 (49)	0.075		0.932	
Gender					
Male	170 (80)	0.708		0.603	
Female	43 (20)	0.839		0.372	
**Mean tumor size** (cm)					
≤ 2.5	134 (63)	0.741		0.139	
> 2.5	79 (37)	0.789		0.125	
**Histological grade**					
Low	94 (44)	0.349		** < 0.0001**	
High	119 (56)	0.554		** < 0.0001**	
pT stage					
pTa	87 (40.8)				
pT1	95 (44.6)	0.219		** < 0.0001**	
pT2	31 (14.6)	0.579		** < 0.0001**	
pT3	0 (0)				
pT4	0 (0)				
**Lamina propria involvement**					
Involved	126 (59)	0.292		** < 0.0001**	
None	87 (41)	0.954		** < 0.0001**	
**Muscularis invasion**					
Involved	31 (14.6)	0.14		0.334	
None	182 (85.4)	0.339		0.684	
**Lamina propria/muscularis involvement (L/M)**					
L − /M-	87 (40.8)	0.219			
L + /M-	95 (44.6)	0.579		**0.0001**	
L + /M +	31 (41.6)			** < 0.0001**	
Recurrence					
Present	57 (27)	0.577		0.203	
Absent	156 (73)	0.692		0.695	
**Distant metastasis**					
Present	33 (15.5)	0.262		0.097	
Absent	180 (84.5)	0.49		0.932	

Bold numbers represent significant *p* values.

Finally, the data in Table 2 did not display correctly. The original Table 2 and accompanying legend appear below.

**Table 2:** Association between MAGE-A11 expressions (staining intensity and H-score) and clinic-pathological parameters of BC cases (*p* value, Pearson’s chi-square test).

Patients and tumor characteristics	Total samples N (%)	Nuclear expression of MAGE-A11		Cytoplasmic expression of MAGE-A11	
		Staining Intensity	H-score	Staining Intensity	H-score
**Median age**					
**Years**					
≤ 67	108 (51)	0.079	0.075	0.638	0.932
> 67	105 (49)				
Gender					
Male	170 (80)	0.708	0.839	0.603	0.372
Female	43 (20)				
**Mean tumor size (cm)**					
≤ 2.5	134 (63)	0.741	0.789	0.139	0.125
> 2.5	79 (37)				
**Histological grade**					
Low	94 (44)	0.349	0.554	** < 0.0001**	** < 0.0001**
High	119 (56)				
pT stage					
pTa	87 (40.8)				
pT1	95 (44.6)	0.219	0.579	** < 0.0001**	** < 0.0001**
pT2	31 (14.6)				
pT3	0 (0)				
pT4	0 (0)				
**Lamina propria involvement**					
Involved	126 (59)	0.292	0.954	** < 0.0001**	** < 0.0001**
None	87 (41)				
**Muscularis invasion**					
Involved	31 (14.6)	0.14	0.339	0.334	0.684
None	182 (85.4)				
**Lamina propria/muscularis involvement (L/M)**					
L − /M-	87 (40.8)				
L + /M-	95 (44.6)	0.219	0.579	**0.0001**	** < 0.0001**
L + /M +	31 (41.6)				
Recurrence					
Present	57 (27)	0.577	0.692	0.203	0.695
Absent	156 (73)				
**Distant metastasis**					
Present	33 (15.5)	0.262	0.49	0.097	0.932
Absent	180 (84.5)				

Bold numbers represent significant *p* values.

The original Article has been corrected.

