# Supplementary material for: Co-expression of cancer-testis antigens of MAGE-A6 and MAGE-A11 is associated with tumor aggressiveness in patients with bladder cancer
Source: Sci Rep. 2022 Jan 12;12:599. doi: 10.1038/s41598-021-04510-2 (PMC8755713; doi:10.1038/s41598-021-04510-2)
Supplement: Supplementary file 1 — Supplementary Figure 1. [file 41598_2021_4510_MOESM1_ESM.pdf]

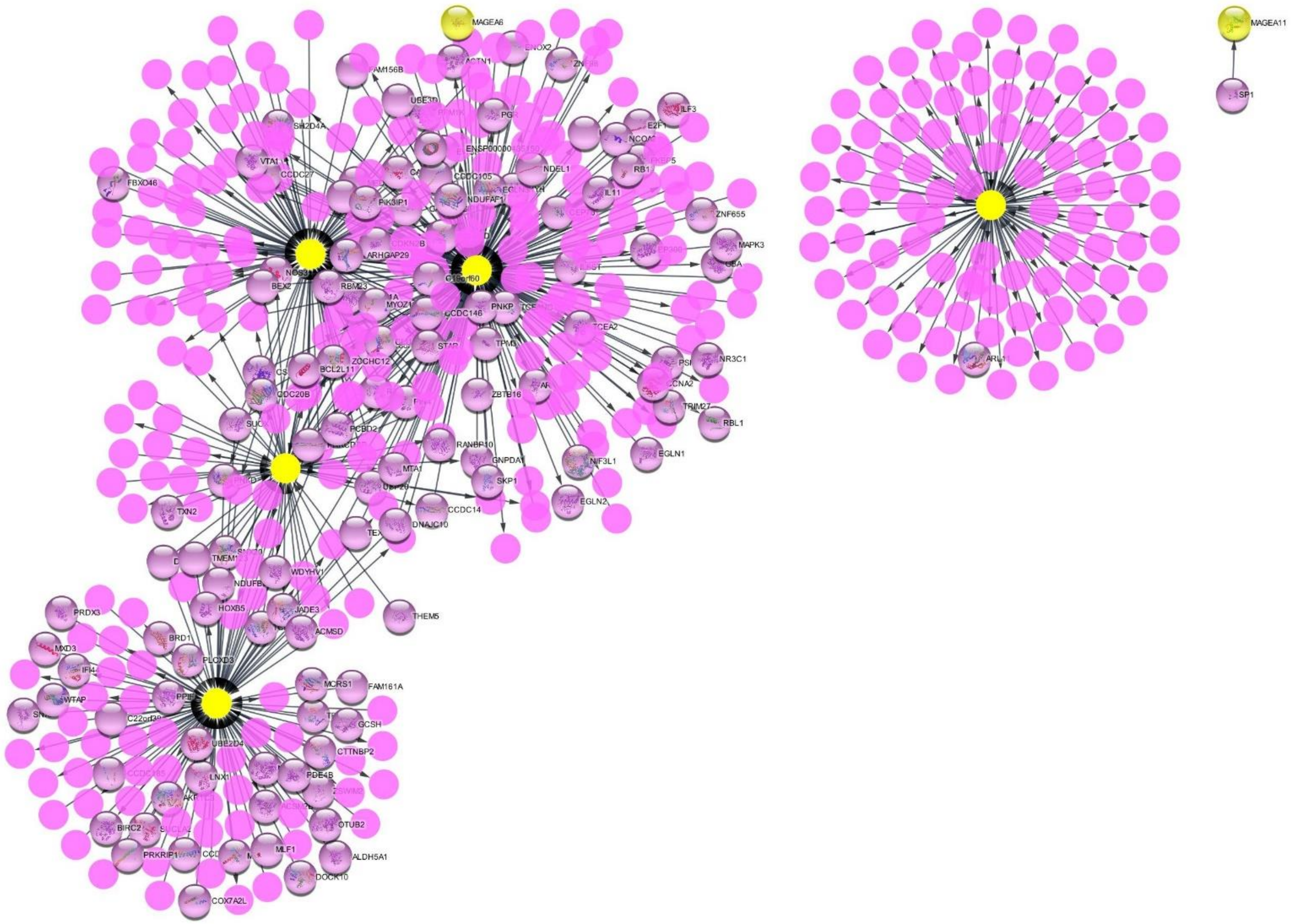

MAGEA6
MAGEA11
SP1
AM156B
UBE3D
SH2D4A
VTA1
FBXO46
CCDC27
ARHGAP29
BEX2
RBM23
ENSP00000
NDEL1
NCOA
E2F1
RB1
IL11
ILF3
ZNF655
MAPK3
CCDC105
PIK3IP1
NDUFAF1
CCDC146
PNKP
TCEA2
TPM3
STAP
ZBTB16
NR3C1
CCNA2
TRIM27
RBL1
EGLN1
EGLN2
NIF3L1
SKP1
GNPDA1
RANBP10
MTA1
CCDC14
DNAJC10
ZCCHC12
BCL2L11
CDC20B
PCBD2
TXN2
WDYHV1
NDUFB
HOXB5
JADE3
ACMSD
THEM5
PRDX3
BRD1
MXD3
PLCXD3
WTAP
MCRS1
FAM161A
GCSH
CTTNBP2
UBE2D4
LNX1
PDE4B
OTUB2
BIRC2
PRKRIP1
MLF1
ALDH5A1
DOCK10
COX7A2L
ARL11

Supplementary Figure 1: The Pathway analysis usage of cytoscape ClueGO plugin (https://cytoscape.org/) (1,2).

The ClueGO plugin visualize to identify enriched pathways amongst all statistically significant proteins. Nodes containing proteins from related KEGG terms are colored the same. The crosslink figure of pathways using ClueGO indicate several genes such as MAGE-A6 and MAGE-A11 are interconnected to BC samples. This bladder cancer related network genes were designed based on TCGA dataset".

1.Shannon P, Markiel A, Ozier O, Baliga NS, Wang JT, Ramage D, et al. Cytoscape: a software environment for integrated models of biomolecular interaction networks. Genome Res. 2003;13(11):2498-504.

2.Bindea G, Mlecnik B, Hackl H, Charoentong P, Tosolini M, Kirilovsky A, et al: a Cytoscape plug-in to decipher functionally grouped gene ontology and pathway annotation networks. Bioinformatics. 2009 ;25(8):1091-3.
