# Supplementary material for: Co-expression of cancer-testis antigens of MAGE-A6 and MAGE-A11 is associated with tumor aggressiveness in patients with bladder cancer
Source: Sci Rep. 2022 Jan 12;12:599. doi: 10.1038/s41598-021-04510-2 (PMC8755713; doi:10.1038/s41598-021-04510-2)
Supplement: Supplementary file 2 — Supplementary Figure 2. [file 41598_2021_4510_MOESM2_ESM.pdf]

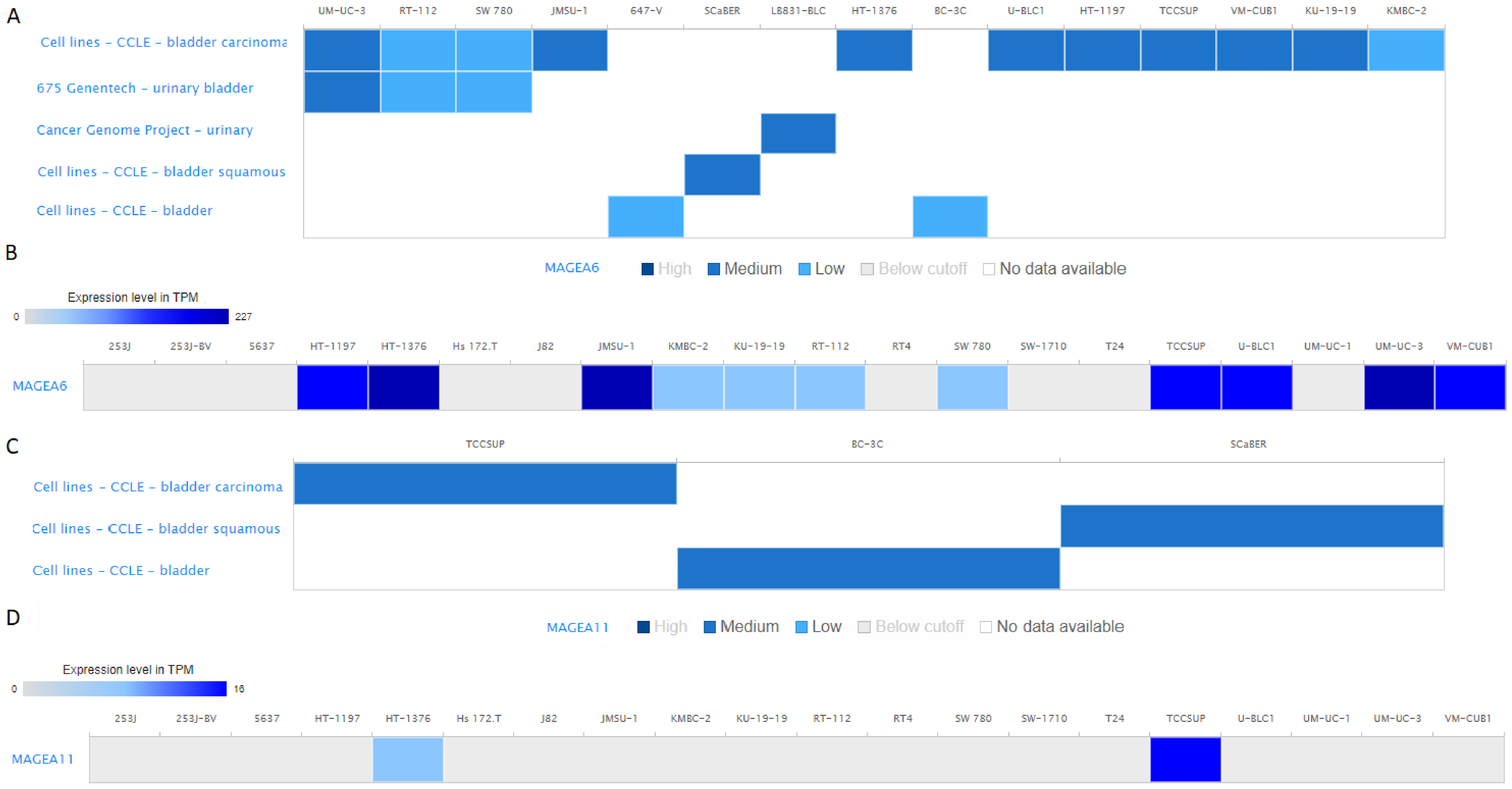

A
UM-UC-3
RT-112
SW 780
JMSU-1
647-V
SCaBER
LB831-BLC
HT-1376
BC-3C
U-BLC1
HT-1197
TCCSUP
VM-CUB1
KU-19-19
KMBC-2
Cell lines - CCLE - bladder carcinoma
675 Genentech - urinary bladder
Cancer Genome Project - urinary
Cell lines - CCLE - bladder squamous
Cell lines - CCLE - bladder
B
MAGEA6
High
Medium
Low
Below cutoff
No data available
Expression level in TPM
0
227
253J
253J-BV
5637
HT-1197
HT-1376
Hs 172.T
J82
JMSU-1
KMBC-2
KU-19-19
RT-112
RT4
SW 780
SW-1710
T24
TCCSUP
U-BLC1
UM-UC-1
UM-UC-3
VM-CUB1
MAGEA6
C
TCCSUP
BC-3C
SCaBER
Cell lines - CCLE - bladder carcinoma
Cell lines - CCLE - bladder squamous
Cell lines - CCLE - bladder
D
MAGEA11
High
Medium
Low
Below cutoff
No data available
Expression level in TPM
0
16
253J
253J-BV
5637
HT-1197
HT-1376
Hs 172.T
J82
JMSU-1
KMBC-2
KU-19-19
RT-112
RT4
SW 780
SW-1710
T24
TCCSUP
U-BLC1
UM-UC-1
UM-UC-3
VM-CUB1
MAGEA11

Supplementary Figure 2: Expression atlas (EMBL-EBI's resource) for MAGE-A6 and MAGE-A11 gene expression in bladder cancer cell lines. **(A)** the medium baseline expression of MAGE-A6 can be found in UM-UC-3, JMSU-1, SCaBER, LB831-BLC, HT-1376, U-BLC1, HT-1197, TCCSUP, VM-CUB1, and KU-19-19 bladder cancer cell lines in five different experiments, **(B)** the high baseline expression of MAGE-A6 can be declared in HT-1376, JMSU-1, and UM-UC-3 cell lines and lower expression in HT-1197, TCCSUP, U-BLC1, VM-CUB1, sequentially, **(C)** the medium baseline expression of MAGE-A11 can be identified in TCCSUP, BC-3C, and SCaBER cell lines in three different experiments, **(D)** the medium baseline expression of MAGE-A11 was shown in TCCSUP cell line.
